# Genotype–phenotype relationship and comparison between eastern and western patients with osteogenesis imperfecta

**DOI:** 10.1007/s40618-023-02123-2

**Published:** 2023-06-04

**Authors:** X. Lin, J. Hu, B. Zhou, Q. Zhang, Y. Jiang, O. Wang, W. Xia, X. Xing, M. Li

**Affiliations:** Department of Endocrinology, National Health Commission Key Laboratory of Endocrinology, Peking Union Medical College Hospital, Chinese Academy of Medical Sciences and Peking Union Medical College, Beijing, 100730 China

**Keywords:** Osteogenesis imperfecta, Phenotype–genotypic correlation, East‒West comparison

## Abstract

**Purpose:**

To evaluate the genotypic and phenotypic relationship in a large cohort of OI patients and to compare the differences between eastern and western OI cohorts.

**Methods:**

A total of 671 OI patients were included. Pathogenic mutations were identified, phenotypic information was collected, and relationships between genotypes and phenotypes were analyzed. Literature about western OI cohorts was searched, and differences were compared between eastern and western OI cohorts.

**Results:**

A total of 560 OI patients were identified as carrying OI pathogenic mutations, and the positive detection rate of disease-causing gene mutations was 83.5%. Mutations in 15 OI candidate genes were identified, with *COL1A1* (*n = *308, 55%) and *COL1A2* (*n = *164, 29%) being the most common mutations, and *SERPINF1* and *WNT1* being the most common biallelic variants. Of the 414 probands, 48.8, 16.9, 29.2 and 5.1% had OI types I, III, IV and V, respectively. Peripheral fracture was the most common phenotype (96.6%), and femurs (34.7%) were most commonly affected. Vertebral compression fracture was observed in 43.5% of OI patients. Biallelic or *COL1A2* mutation led to more bone deformities and poorer mobility than *COL1A1* mutation (all *P* < 0.05). Glycine substitution of *COL1A1* or *COL1A2* or biallelic variants led to more severe phenotypes than haploinsufficiency of collagen type I α chains, which induced the mildest phenotypes. Although the gene mutation spectrum varied among countries, the fracture incidence was similar between eastern and western OI cohorts.

**Conclusion:**

The findings are valuable for accurate diagnosis and treatment of OI, mechanism exploration and prognosis judgment. Genetic profiles of OI may vary among races, but the mechanism needs to be explored.

**Supplementary Information:**

The online version contains supplementary material available at 10.1007/s40618-023-02123-2.

## Introduction

Osteogenesis imperfecta (OI) is a phenotypically and genotypically heterogeneous connective tissue disorder, with an incidence of one in 15 000–20 000 births [1]. OI is a fairly common genetic bone disorder characterized by low bone mineral density (BMD), recurrent fractures, progressive bone deformity and extraskeletal manifestations, such as blue sclerae, dentinogenesis imperfecta, hearing loss and joint laxity [2]. The manifestations of OI are diverse, and the several different forms of OI are associated with considerable morbidity and mortality [3].

OI is mainly caused by mutations in *COL1A1* and *COL1A2*, encoding genes of the α1(I) and α2(I) chains of type I collagen, which lead to structural or quantitative defects of the essential bone extracellular matrix protein type I collagen [4]. Recently, autosomal recessive forms and X-linked OI have been identified, which are caused by mutations in multiple genes participating in folding or posttranslational modifications of type I collagen, osteoblast differentiation, or bone mineralization [5]. With advances in molecular investigations, a total of 24 genes have been identified as the pathogenic genes of OI, including *COL1A1*, *COL1A2*, *CRTAP*, *FKBP10*, *PLOD2*, *P3H1*, *PPIB*, *SERPINF1*, *SERPINH1*, *SP7*, *WNT1*, *BMP1*, *TMEM38B*, *IFITM5*, *PLS3*, *CREB3L1*, *SEC24D*, *SPARC*, *P4HB*, *MBTPS2*, *KDELR2*, *FAM46A, MESD* and *CCDC134* [1]. Currently, over 2000 variants have been reported (HGMD, www.hgmd.cf.ac.uk, accessed Sept. 3, 2022), leading to a variety of skeletal and extraskeletal phenotypes [6].

OI is not rare in China because of the large population. Annually, approximately 500–700 Chinese children are born with OI. [7]. Although previous studies have described the relationship of phenotypes and genotypes in OI patients, an in-depth study regarding genotypic and skeletal phenotypic relationships in a large cohort of Asian OI patients has not yet been reported [8–11]. Additionally, it remains unclear whether differences in phenotypes and genotypes exist between Eastern and Western OI patients. Therefore, we aimed to investigate the genotypes and phenotypes of a large cohort of OI, to explore their relationship, and to compare differences in genotypes and phenotypes between Eastern and Western OI cohorts.

## Methods

### Subjects

Patients were enrolled at the Endocrinology Department of Peking Union Medical College Hospital (PUMCH) from January 2007 to May 2022. Eligible patients were clinically suspected of having OI on the basis of a history of fracture under minor force, low BMD, with or without blue sclera, dentinogenesis imperfecta or a familial history of OI or fracture.

This study was approved by the Scientific Ethics Committee of PUMCH (JS-3545D). Informed consent was obtained from the patients or their legal guardians before participation in the study.

### Phenotypic evaluation

Clinical data for the patients were collected from medical records, including birth situation, age at diagnosis, age of initial fracture, fracture frequency and site, bone deformities (limb bending, thoracic deformity, and pelvic deformity), and extraskeletal manifestations. Body height and weight were measured using a Harpenden stadiometer (Seritex Inc., East Rutherford, NJ, USA). For patients unable to stand, body length was measured in the supine position. The height of all OI patients was converted to standard deviation score (SDS) using standardized growth charts for Chinese children and adolescents [12]. OI was classified into five subtypes according to the clinical severity: mild OI (type I), perinatally lethal OI (type II), progressive deforming OI (type III), intermediate OI (type IV) and OI with hypertrophic callus (type V) [5].

BMDs at the lumbar spine (LS) and proximal hip were measured by dual-energy X-ray absorptiometry (DXA, Lunar Prodigy Advance, GE Healthcare, USA), and appropriate pediatric software was used for measurement of BMD in children. A quality control program was conducted throughout the study, and phantom testing was completed daily using the DXA device for calibration and quality checks. Obviously compressed or deformed vertebrae were excluded from BMD analysis. LS and FN BMD results of children and adolescents were converted to age- and sex-specific Z scores according to normal reference of BMD in Asian children [13, 14].

Serum levels of β-isomerized carboxy-telopeptide of type I collagen (β-CTX, a bone resorption marker), procollagen I N-terminal peptide (P1NP, a bone formation marker), 25-hydroxyvitamin D (25OHD) and intact parathyroid hormone (PTH) were measured using an automated electrochemiluminescence system (E170, Roche Diagnostics, Switzerland). Serum levels of alanine aminotransferase (ALT), creatinine (Cr), calcium (Ca), phosphate (P) and alkaline phosphatase (ALP, a bone formation marker) were assessed using automated analyzers (ADVIA 1800, Siemens, Germany). All parameters were detected in the clinical laboratory of PUMCH.

### Bone fracture and scoliosis evaluation

Clinical fractures were reported by the patients or their legal guardians and confirmed by X-ray films, including nonvertebral fractures and symptomatic vertebral fractures. Vertebral compression fracture (VCF) was semiquantitatively assessed as normal (grade 0), mildly deformed (grade 1), moderately deformed (grade 2), and severely deformed (grade 3) according to Genant's classification [15]. Scoliosis was determined by anteroposterior radiography and defined as a Cobb angle > 10 degree [16]. X-ray film results were interpreted by radiologists at PUMCH.

### Genotypic analysis

Total genomic DNA was isolated from peripheral blood using a DNA Extraction Mini Kit (QIAamp DNA, Qiagen, Frankfurt, Germany). Clinically diagnosed OI patients underwent panel sequencing (Illumina HiSeq2000 platform, Illumina Inc., San Diego, CA, USA) using a previously described protocol [17]. The next-generation sequencing (NGS) panel covers more than 700 candidate genes of disorders related to bone, including 20 known candidate genes for OI (*COL1A1*, *COL1A2*, *IFITM5*, *SERPINF1*, *CRTAP*, *P3H1*, *PPIB*, *SERPINH1*, *FKBP10*, *PLOD2*, *BMP1*, *SP7*, *TMEM38B*, *WNT1*, *CREB3L1, SPARC*, *MBTPS2*, *P4HB*, *SEC24D* and *PLS3*). The overall sequencing coverage of the target regions was more than 95% at a minimum sequencing depth of 20 ×  Bioinformatics processing and data analysis were performed. The “clean reads” derived from targeted sequencing and filtering were aligned to the human genome reference (hg19) using the BWA (Burrows Wheeler Aligner) Multi-Vision software package. All SNVs and indels were filtered and estimated with multiple databases (NCBI dbSNP, HapMap, 1000 human genome dataset and database of 100 Chinese healthy adults). The deleterious effects of missense variants on the corresponding proteins were predicted by silico tools (MutationTaster, PolyPhen-2, SIFT and PhyloP). Variants were classified according to the 2015 American College of Medical Genetics and Genomics/Association for Molecular Pathology (ACMG/AMP) guidelines [18].

Pathogenic variants identified by NGS were confirmed by Sanger sequencing. Targeted primers were designed, PCR was performed, and the amplicons generated using the primers designed were sequenced with a 3730 DNA analyzer (Applied Biosystems, Foster City, CA, USA). Segregation analysis was performed if DNA was available from the family members. *COL1A1* and *COL1A2* variants were categorized based on the effects of gene mutation on type I collagen synthesis as glycine substitution, nonglycine substitution, haploinsufficiency or splicing mutation [19]. Compound heterozygous or homozygous mutation patterns were classified as biallelic variants [20].

### Comparison between eastern and western OI cohorts

We searched for previously published large cohorts of OI from Western and Eastern countries in PubMed, Embase, and Medline databases using “osteogenesis imperfecta”, “cohort”, “children with bone fracture”, and “children with osteoporosis”. We collected data on age at OI diagnosis, family history, height, fracture frequency, BMDs, classification of OI and gene mutations in these cohorts of OI, and differences between Eastern and Western OI cohorts were compared.

### Statistical analysis

Continuous variables were tested for normal distribution using the Kolmogorov‒Smirnov test. Normally distributed data (BMD Z-scores) are presented as the mean ± standard deviation (SD). Differences in BMD Z-scores between groups were compared with one-way ANOVA. Nonnormally distributed data are expressed as medians (range), including age, age at first fracture, height Z-score, frequency of peripheral fracture, and serum PTH and 25OHD levels, and the Mann‒Whitney U test was used to compare these parameters between groups. Categorical data are presented as frequencies and percentages (%), Fisher's exact test was utilized to compare these categorical variables (positive family history of fracture, peripheral fracture, VCF, long bone deformity, scoliosis, etc.) between groups. The association between the position of glycine substitution and the frequency of peripheral fracture, LS and FN BMD Z-score, or height Z-score was evaluated using Spearman rank correlation coefficient analysis.

Statistical analyses were performed using SPSS software (version 26.0; SPSS Inc., Chicago, IL, USA). A two-tailed value of *P* < 0.05 was considered statistically significant.

## Results

### Gene mutation spectrum

**Fig. 1:**
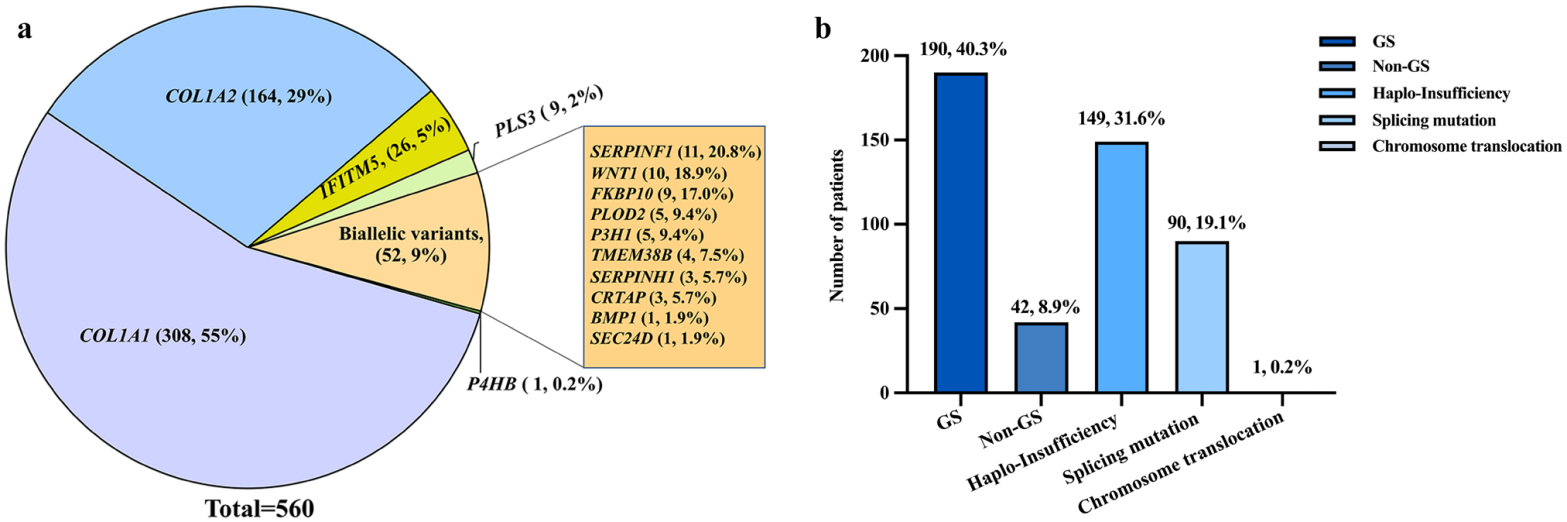
Mutation spectrum of this largest Chinese OI cohort. **a** Mutation spectrum of all OI patients. **b** Mutation types of *COL1A1* and *COL1A2* in this cohort. *OI* osteogenesis imperfecta

A total of 671 patients with a clinical diagnosis of OI were included in this study. Of these, 560 patients from 427 unrelated families were identified as carrying OI pathogenic mutations, and the rate of disease-causing gene mutation positivity was 83.5%. The pathogenic mutations of 427 probands identified in this cohort are listed in Supplementary Table 1. Mutations in 15 OI candidate genes were identified, with *COL1A1* (*n = *308, 55%) and *COL1A2* (*n = *164, 29%) being the most common (Fig. 1a). Among these mutations, 275 (49%) are missense, 69 (12%) nonsense, 118 (21%) frameshift, 101 (18%) splicing and 1 (0.2%) a chromosome translocation. Among the *COL1A1/COL1A2* variants (*n = *472), 190 (40.3%) are glycine substitutions, 42 (8.9%) nonglycine substitutions, 149 (31.6%) haploinsufficiency, 90 (19.1%) splicing mutations and 1 (0.2%) a chromosome translocation (Fig. 1b). Additionally, c.769G > A (p.G257R) of *COL1A1* and c.1009G > A (p.G337S) of *COL1A2*, which were the most common variants of *COL1A1* and *COL1A2*, were identified in 10 and 6 unrelated families, respectively, suggesting that these two mutations are hotspot mutations in the Chinese OI cohort. Among biallelic variants, mutations in *SERPINF1* (21.2%) and *WNT1* (19.2%) were most frequent (Fig. 1a).

### Phenotypic spectrum

**Fig. 2:**
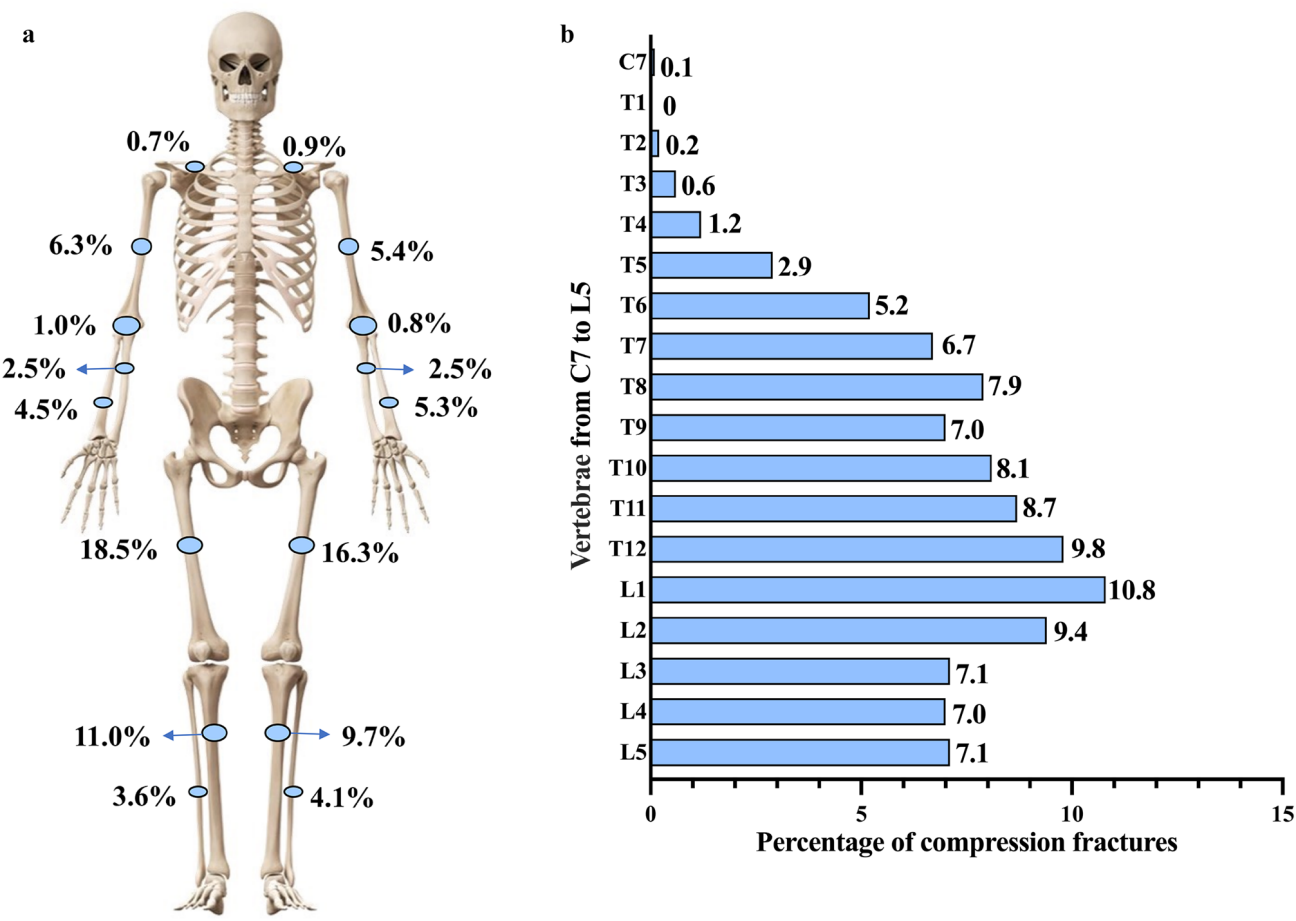
Association of gene mutation with clinical severity of OI. **a** Association between different mutated genes and the clinical severity of OI. **b** Association between different collagen changes induced by gene mutations and clinical severity of OI. *OI* osteogenesis imperfecta, *GS* glycine substitution

Detailed clinical data were available for 414 OI probands, including 328 children and 86 adults (Table 1). A total of 197 patients (48.3%) had a positive family history of OI. The median age of initial visit and age at first fracture among the OI patients were 10.0 years (range: 0.2–64.0 years) and 1.5 years (range: 0.0–59.0 years), respectively. The most common clinical manifestation was peripheral fracture (96.6%), and total frequency varied greatly, ranging from 0 to 70 times, with a median time of 4.0. (Table 1). A total of 2887 peripheral fractures were noted in this cohort, with femurs being the most commonly affected sites (34.8%). (Fig. 2a). VCF was also common in this study, as noted in 180 (43.5%) OI patients. A total of 815 VCFs were recorded from C7 to L5, with the most common sites at L1 (10.8%) and T12 (9.8%) (Fig. 2b). Overall, 251 OI patients (60.6%) developed bone deformities, including long bone and ribcage deformities and scoliosis (Table 1). Based on clinical phenotype, 202 (48.8%) patients were classified as OI type I, 70 (16.9%) as OI type III, 121 (29.2%) as OI type IV, and 21 (5.1%) as OI type V (Table 1). Additionally, among 111 patients with no detectable OI mutations, 81 (73.0%) were classified as OI type I, 9 (8.1%) as OI type III and 21 (18.9%) as OI type IV.

**Table 1:** Demographic and clinical characteristics of patients with OI

	Children *n = *328	Adults *n = *86	Total *n = *414	Reference range
Age of initial visit (years)	6.6 (0.2–17.7)	28.7 (18.0–64.0)	10.0 (0.2–64.0)	
Age at first fracture, (years)	1.5 (0.0–13.0)	2.0 (0.0–59.0)	1.5 (0.0–59.0)	
Positive family history, *n* (%)	154 (47.0)	43 (50.0)	197 (48.3)	
Sillence type, I/III/IV, *n*	171/38/101	31/32/20	202/70/121	
Height Z-score	− 1.0 (− 17.1–5.4)	− 2.5 (− 19.5–2.1)	− 1.2 (− 19.5–5.4)	
Peripheral fracture, *n* (%)	315 (96.0)	85 (98.8)	400 (96.6)	
Frequency of peripheral fracture				
Total	4.0 (0.0–60.0)	10.0 (1.0–70.0)	4.0 (0.0–70.0)	
Per year	1.0 (0.0–15.3)	0.4 (0.0–3.7)	0.9 (0.0–15.3)	
VCF, *n* (%)	139 (42.4)	41 (47.7)	180 (43.5)	
Grade I	62 (44.6)	17 (41.5)	79 (43.9)	
Grade II	23 (16.5)	5 (12.2)	28 (6.8)	
Grade III	31 (22.3)	11 (26.8)	42 (10.1)	
Unknown	23 (16.5)	8 (19.5)	31 (17.2)	
Wheelchair dependence, *n* (%)	123 (37.5)	36 (41.9)	159 (38.4)	
Long bone deformity, *n* (%)	163 (49.7)	46 (53.5)	209 (50.5)	
Scoliosis, *n* (%)	60 (18.3)	43 (50.0)	103 (24.9)	
Ribcage deformity, *n* (%)	31 (9.5)	23 (26.7)	54 (13.0)	
Blue sclera, *n* (%)	268 (81.7)	63 (73.3)	331 (80.0)	
Dentinogenesis imperfecta, *n* (%)	58 (17.7)	18 (20.9)	76 (18.4)	
Ligament laxity, *n* (%)	153 (46.6)	37 (43.0)	190 (45.9)	
Hearing abnormality, *n* (%)	5 (1.5)	14 (16.3)	19 (4.6)	
Thin long bone cortex, *n* (%)	214 (65.2)	38 (44.2)	252 (60.9)	
Wormian bone, *n* (%)	202 (61.6)	43 (50.0)	245 (59.2)	
ALT, U/L	14.0 (4.0–275.0)	16.0 (6.0–124.0)	14.0 (4.0–275.0)	7–40
Cr, μmol/L	32.0 (4.0–76.0)	52.5 ± 13.7	36.0 (4.0–95.0)	45–84
ALP, U/L	295.5 ± 101.5	94.9 ± 33.4	254.4 ± 122.2	adults: 35–100 children: 42–390 [43]
Ca, mmol/L	2.5 ± 0.1	2.4 ± 0.1	2.5 ± 0.1	2.13–2.70
P, mmol/L	1.7 ± 0.2	1.2 ± 0.2	1.6 ± 0.3	adults: 0.81–1.45 children: 0.95–2.65 [43]
P1NP, ng/mL	370.8 (55.0–1082.0)	37.8 (6.5–263.1)	312.1 (6.5–1082.0)	adults: 15.1–58.6 children: 30.0–3000.0 [44]
β-CTX, ng/mL	0.9 ± 0.4	0.3 (0.02–1.05)	0.8 ± 0.4	adults: 0.21–0.44 children: 0.40–3.30 [44]
PTH, pg/mL	22.3 (3.0–153.1)	39.5 (12.3–108.1)	25.3 (3.0–153.10)	15.0–65.0
25OHD, ng/mL	22.6 (6.0–92.2)	15.8 (6.6–79.0)	20.9 (6.0–92.2)	> 30
LS BMD Z-score	− 2.2 ± 1.8	− 2.2 ± 1.5	− 2.2 ± 1.8	
FN BMD Z-score	− 3.5 ± 2.1	− 1.7 ± 1.8	− 3.1 ± 2.12	

Normally distributed results are given as the mean ± SD

Nonnormally distributed results are given as the median (range)

Categorical data are presented as frequencies (%)

*ALP* alkaline phosphatase, *ALT* glutamic-pyruvic transaminase, *Ca* calcium, *Cr* creatinine, *FN* femoral neck, *LS* lumbar spine, *OI* osteogenesis imperfecta, *P* phosphorus, *P1NP* procollagen type 1 N-peptide, *PTH* parathyroid hormone, *β-CTX* β-C-terminal telopeptide of type 1 collagen, *25OHD* 25-hydroxyvitamin D, *VCF* vertebral compression fracture

Bold numbers represent *P*** < **0.05

Additionally, according to serum 25OHD level, vitamin D sufficiency (25OHD level > 30 ng/mL), insufficiency (21–29 ng/mL) and deficiency (< 20 ng/mL) were found in 22.9% (80/349), 29.8% (104/349) and 47.3% (165/349) of OI patients, respectively. A total of 5.2% (18/349) of OI patients exhibited secondary hyperparathyroidism caused by insufficiency or deficiency of vitamin D. Furthermore, the levels of 25OHD were negatively correlated with age of OI patients (Supplementary table 2 and Supplementary Fig. 1).

### Relationships between genotypes and phenotypes

**Fig. 3:**
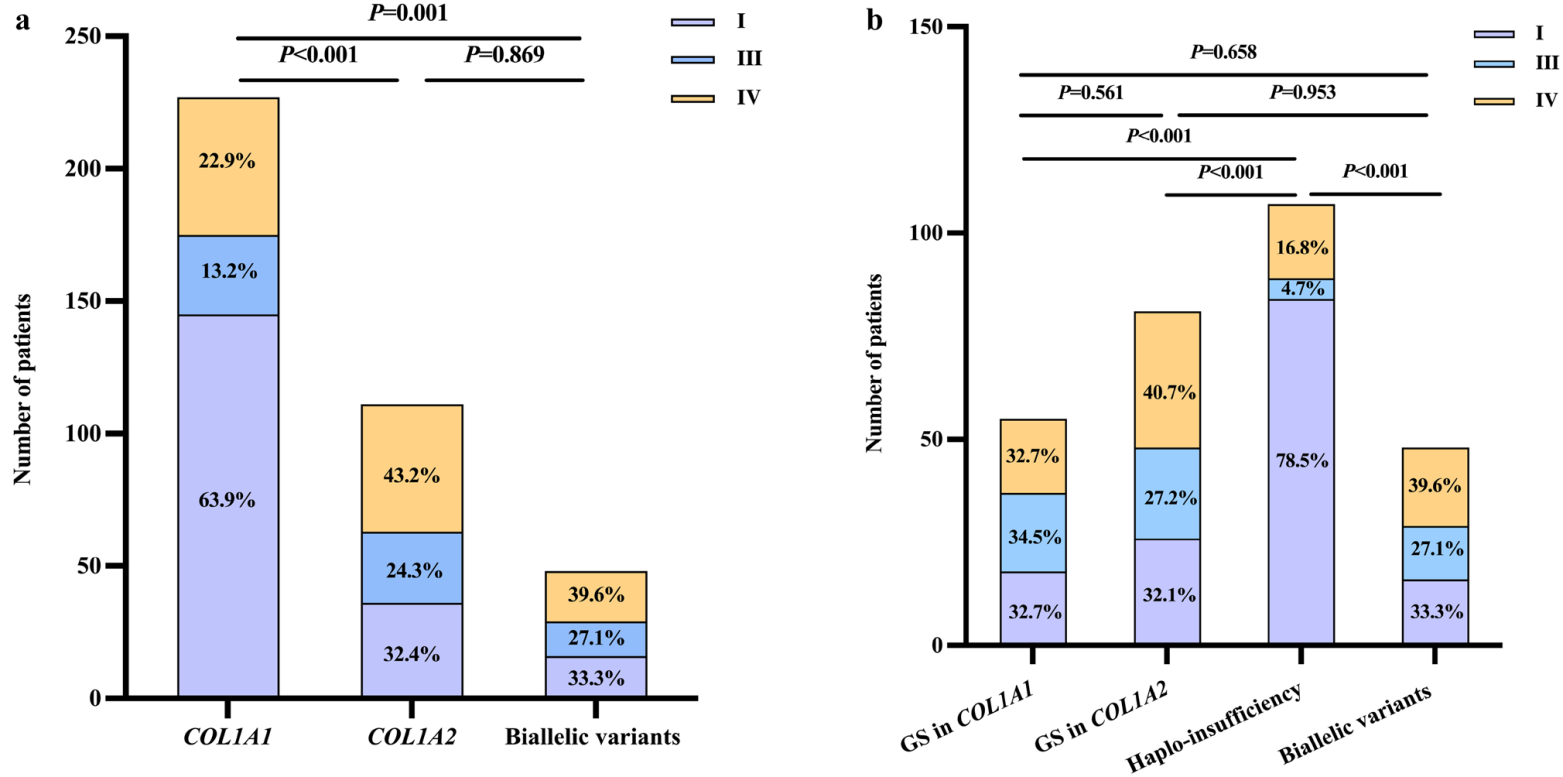
Distribution of fracture sites in OI patients. **a** Distribution of fracture sites in OI patients with peripheral bone fractures, **b** Distribution of fracture sites in OI patients with VCFs. *OI* osteogenesis imperfecta, *VCF* vertebral compression fracture

Phenotypic differences in OI patients with *COL1A1*, *COL1A2*, and biallelic mutations were compared. Patients carrying *COL1A1* mutation showed the mildest skeletal phenotypes, whereas those carrying *COL1A2* or biallelic mutation had more severe phenotypes, including shorter height Z-score (− 0.8 vs. − 2.2, *P* < 0.001; − 0.8 vs. − 1.6, *P* = 0.004), more long bone deformity (41.9% vs. 60.4%, *P* = 0.001; 41.9% vs. 59.2%, *P* = 0.027) and ribcage deformity (7.0% vs. 17.1%, *P* = 0.004; 7.0% vs. 26.5%, *P* < 0.001), poorer mobility (31.3% vs. 45.9%, *P* = 0.008; 31.3% vs. 57.1%, *P* = 0.001) and higher OI type III frequency (13.2% vs. 24.3%, *P* = 0.010; 13.2% vs. 26.5%, *P* = 0.020) (Table 2, Fig. 3a).

**Table 2:** Comparison of phenotypes of OI patients with different genotypes

	*COL1A1* mutation *n = *227	*COL1A2* mutation *n = *111	Biallelic variants *n = *48	*P* value
Age of initial visit (years)	9.2 (0.2–64.0)	11.3 (1.0–62.0)	9.0 (1.8–39.0)	0.336
Age at first fracture (years)	1.5 (0.0–59.0)	1.1 (0.0–19.0)	1.7 (0.0–37.0)	0.360
Positive family history, *n* (%)	104 (45.8)	53 (47.7)	11 (22.9) ^c,d^	**0.006**
Height Z-score	− 0.8 (− 12.1–5.4)	− 2.2 (− 19.5–3.4)^a^	− 1.6 (− 12.1–1.5)^c^	** < 0.001**
Limb fracture, *n* (%)	217 (95.6)	108 (97.3)	47 (97.9)	0.729
Frequency of peripheral fracture				
Total	4.0 (0.0–60.0)	5.0 (0.0–50.0)^c^	5.0 (0.0–70.0)	**0.013**
Per year	0.9 (0.0–5.3)	0.9 (0.0–15.3)	1.0 (0.0–14.0)	0.621
VCF, *n* (%)	93 (41.0)	45 (40.5)	25 (52.1)	0.408
Grade I	46 (49.5)	14 (31.1)	12 (48.0)	0.121
Grade II	16 (17.2)	10 (22.2)	1 (4.0)	0.129
Grade III	21 (22.6)	9 (20.0)	5 (20.0)	0.931
Unknown	10 (10.8)	12 (26.7)^c^	7 (28.0)^c^	**0.021**
Wheelchair dependence, *n* (%)	71 (31.3)	51 (45.9)^c^	27 (56.3)^c^	**0.001**
Long bone deformity, *n* (%)	95 (41.9)	67 (60.4)^c^	33 (68.8)^c^	**0.002**
Scoliosis, *n* (%)	49 (21.6)	25 (22.5)	19 (39.6)^c,d^	**0.015**
Ribcage deformity, *n* (%)	16 (7.0)	19 (17.1)^c^	13 (27.1)^a^	** < 0.001**
LS BMD Z-score	− 2.0 ± 1.7	− 2.6 ± 1.6^c^	− 2.5 ± 2.4	**0.003**
FN BMD Z-score	− 2.9 ± 1.9	− 3.8 ± 2.4^a^	− 3.2 ± 2.2	** < 0.001**

Normally distributed results are given as the mean ± SD. Nonnormally distributed results are given as the median (range). Categorical data are presented as frequencies (%)

*FN* femoral neck, *LS* lumbar spine, *OI* osteogenesis imperfecta, *VCF* vertebral compression fracture

^a^*P* < 0.001 compared to *COL1A1* mutation; ^b^*P* < 0.001 compared to *COL1A2* mutation

^c^*P* < 0.05 compared to *COL1A1* mutation; ^d^*P* < 0.05 compared to *COL1A2* mutation

Bold numbers represent *P*** < **0.05

Furthermore, phenotypic differences in OI patients with glycine substitution of *COL1A1* or *COL1A2*, haploinsufficiency and biallelic variants were compared. Among these 4 subgroups, patients with haploinsufficiency of collagen type I α chains exhibited the mildest skeletal phenotypes. In contrast, individuals with glycine substitution of *COL1A1* or *COL1A2* or biallelic variants showed more severe phenotypes, characterized by lower height Z-score, more long bone and ribcage deformities, poorer mobility and lower BMD (all *P* < 0.05) (Table 3). Patients with glycine substitution of *COL1A1* or *COL1A2* or biallelic variants had similar bone involvement (Fig. 3b).

**Table 3:** Comparison of phenotypes of OI patients with different collagen changes in type I α chains and those with biallelic variants

	Glycine substitution in *COL1A1 n = *55	Glycine substitution in *COL1A2* *n = *81	Haploinsufficiency *n = *107	Biallelic variants *n = *48	*P* value
Age of initial visit (years)	11.0 (0.3–58.0)	12.0 (1.0–42.7)	8.7 (0.2–64.0)	9.0 (1.8–39.0)	0.125
Age at first fracture (years)	1.0 (0.0–14.0) ^c^	1.0 (0.0–13.0)	2.0 (0.0–59.0)	1.7 (0.0–37.0)	**0.004**
Positive family history, *n* (%)	22 (40.0)	40 (49.4)	55 (51.4)	11 (22.9)^b, c^	**0.005**
Height Z-score	− 2.3 (− 12.1–2.0)^c^	− 2.5 (− 19.5–3.4)^c^	− 0.4 (− 7.2–3.0)	− 1.6 (− 12.1–1.5)^c^	** < 0.001**
Limb fracture, *n* (%)	55 (100.0)	80 (98.8)	100 (93.5)	47 (97.9)	0.088
Frequency of peripheral fracture					
Total	5.0 (1.0–60.0)	6.0 (1.0–50.0)^c^	4.0 (0.0–20.0)	5.0 (0.0–70.0)	** < 0.001**
Per year	0.8 (0.1–3.0)	0.9 (0.1–4.5)	0.8 (0.0–5.0)	1.0 (0.0–14.0)	0.406
VCF, *n* (%)	35 (63.6)^b,c^	36 (44.4)	35 (32.7)	25 (52.1)^c^	**0.002**
Grade I	14 (40.0)	12 (33.3)	21 (60.0)	12 (48.0)	0.132
Grade II	6 (17.1)	9 (25.0)	6 (17.1)	1 (4.0)	0.184
Grade III	9 (25.7)	6 (7.4)	7 (20.0)	5 (20.0)	0.822
Unknown	6 (17.1)	9 (11.1) ^c^	1 (2.9)	7 (28.0)^c^	**0.020**
Wheelchair dependence, *n* (%)	23 (41.8)^c^	39 (48.1)^c^	12 (11.2)	27 (56.3)^c^	** < 0.001**
Long bone deformity, *n* (%)	37 (67.3)^c^	51 (63.0)^c^	30 (28.0)	33 (68.8)^c^	** < 0.001**
Scoliosis, *n* (%)	26 (47.3)^b,c^	19 (23.5)	14 (13.1)	19 (39.6)^b,c^	** < 0.001**
Ribcage deformity, *n* (%)	10 (18.2)^c^	15 (18.5)^c^	3 (2.8)	13 (27.1) ^c^	** < 0.001**
LS BMD Z-score	− 2.4 ± 1.9^c^	− 2.7 ± 1.5^c^	− 1.8 ± 1.5	− 2.5 ± 2.4^c^	**0.004**
FN BMD Z-score	− 3.0 ± 1.9	− 3.8 ± 2.2^a,c^	− 2.6 ± 1.9	− 3.2 ± 2.2	**0.002**

Normally distributed results are given as the mean ± SD. Nonnormally distributed results are given as the median (range). Categorical data are presented as frequencies (%)

*FN* femoral neck, *LS* lumbar spine, *OI* osteogenesis imperfecta, *VCF* vertebral compression fracture

^a^*P* < 0.05 compared to glycine substitution in *COL1A1*; ^b^*P* < 0.05 compared to glycine substitution in *COL1A2*; ^c^*P* < 0.05 compared to haploinsufficiency

Bold numbers represent *P*** < **0.05

For glycine substitution of *COL1A1/COL1A2*, there were no obvious correlations between the position of the glycine substitution and phenotype, including total frequency of fracture, LS or FN BMD Z-score, and height Z-score. (Supplementary Fig. 2).

### Comparison between eastern and western OI cohorts

We screened 5 relatively large OI cohorts from Western countries, including Canada (*n = *598) [21], Turkey (*n = *150) [20], Italy (*n = *364) [22], America (*n = *544) [23] and Sweden (*n = *223) [24] (Table 4). The annual fracture rate of OI patients seemed to be similar among Turkish, American, Swedish and Chinese OI cohorts (Table 4), indicating that the phenotypic severity of Eastern and Western OI patients might be roughly similar.

**Table 4:** Comparison of genotypes and phenotypes between Eastern and Western OI cohorts

	Canada (*n = *598)	Turkey (*n = *150)	Italy (*n = *364)	America (*n = *544)	Sweden (*n = *223)	Our cohort (*n = *671)	*P* value
Age at diagnosis (years)	15.1 ± 9.6	NA	NA	12.6 (0–67)	NA	10.0 (0.2–64.0)	NA
Children (years)	NA	*COL1A1/A2* variants: 1.6 (0–18.0); Biallelic variants: 1.05 (0–24.0)	7.27 ± 4.7	NA	3.40 ± 3.23	6.6 (0.2–18.0)	NA
Adults (years)	NA	NA	37.0 ± 12.0	NA	17.51 ± 14.48	29.0 (18.4–64.0)	NA
Positive family history, *n* (%)	246 (44.2)	NA	158 (43.4)	219 (40.3)	NA	197 (48.3)	NA
Height Z-score	− 3.4 ± 3.1	− 2.8 ± 2.6	Mean: − 1.77		− 2.10 ± 2.70	− 1.2 (− 19.5–5.4)	NA
Annual fracture frequency^#^	NA	*COL1A1/A2* variants: 1.0 (0–10.0); Biallelic variants: 2.0 (0.2–10.0)	NA	1.03 ± 2.89	1.14 ± 3.58	0.9 (0.0–15.3)	NA
LS BMD Z-score	NA	NA	NA	NA	− 3.28 ± 1.24	− 2.23 ± 1.77	NA
FN BMD Z-score	NA	NA	NA	NA	NA	− 3.12 ± 2.12	NA
Gene sequencing method	Sanger sequencing, NGS	Sanger sequencing, MLPA, NGS, WES	Sanger sequencing	NA	Sanger sequencing, MLPA	NGS	NA
Genetic testing, *n*	598	140	364	370	216	671	
Positive detection rate, *n* (%)	585 (97.8)	117 (83.6)	309 (84.9)	343 (92.7)	188 (87.0)	560 (83.5)	** < 0.001**
*COL1A1*, *n* (%)	354 (60.5)	52 (44.4)	230 (74.4)	228 (61.6)	149 (79.3)	308 (55.0)	** < 0.001**
*COL1A2*, *n* (%)	159 (27.2)	21 (17.9)	79 (25.6)	99 (26.8)	39 (20.7)	164 (29.0)	0.065
*IFITM5*, *n* (%)	30 (5.1)	1 (0.85)	NA	7 (1.9)	NA	26 (5.0)	**0.023**
*PLS3*, *n* (%)	0	0	NA	0	NA	9 (2.0)	**0.001**
Biallelic variants, *n* (%)	40 (6.8)	42 (35.9)	NA	9 (2.4)	NA	53 (9.0)	** < 0.001**

^#^The mean/median number of fractures per year

*FN* femoral neck, *LS* lumbar spine, *MLPA* multiplex ligation-dependent probe amplification, *NA* not available, *NGS* next genome sequencing, *OI* osteogenesis imperfecta, *WES* whole exome sequencing

Moreover, the methods of genetic testing differed among countries. OI cohorts from Italy and Sweden were identified solely by Sanger sequencing or Sanger sequencing combined multiplex ligation-dependent probe amplification (MLPA) covering *COL1A1/COL1A2* genes. OI cohorts from Canada, Turkey and China were identified by NGS panels or WES, which covered currently known candidate genes of OI. The detection rate of disease-causing gene mutation was similar between Turkish (83.6%), Italian (84.9%), Swedish (87.0%), and Chinese (83.5%) cohorts, though it seemed to be higher in Canadian (97.8*%*) and American (92.7%) cohorts (Table 4). Although the distribution of gene mutations differed among countries, *COL1A1* and *COL1A2* were still the dominant mutation genes in all cohorts. Furthermore, we found that *SERPINF1* and *WNT1* were the most common biallelic pathogenic genes in our cohort (21.2%; 19.2%), which was distinct from studies of Canadian, Turkish and American OI patients. In Canadian and American cohorts, *SERPINF1* and *CRTAP* were the most frequently affected biallelic pathogenic genes; in Turkish cohorts, *FKBP10* and *P3H1* were the most frequently affected genes. Interestingly, we found a notable increase in the identification of biallelic variants from 2015 to 2022, which is attributed to the widespread use of NGS technology.

## Discussion

In this study, genetic mutation and phenotypic profiles were explored in the largest sample of Asian OI patients. The phenotypic spectrum indicated peripheral fracture to be the most common phenotype, especially femoral fracture. VCF was also frequent in OI patients, with the most common sites at the L1 and T12 vertebra. The genotypic spectrum revealed *COL1A1* and *COL1A2* to be the dominant pathogenic mutations, with c.769G > A (p.G257R) of *COL1A1* and c.1009G > A (p.G337S) of *COL1A2* as hotspot mutations. We observed a close correlation between the genotype and phenotype of OI patients, with skeletal phenotypes being mildest in patients with haploinsufficiency of collagen type I α chains. These phenotypes were more severe in patients carrying glycine substitution of *COL1A1/COL1A2* or biallelic mutation, including lower height Z-score, more long bone and ribcage deformities, poorer mobility, and lower BMD. We compared differences in genotype and phenotype for the first time between Asian and Western OI cohorts and found that the annual incidence of fractures was similar, though the gene mutation spectrum among countries differed.

The underlying mechanism of OI is quite complicated, and the fundamental mechanism involves abnormal collagen metabolism induced by multiple gene mutations. There are two general classes of mutations in type I collagen that result in OI: failure of type I collagen synthesis (haploinsufficiency) and structural abnormalities of collagen molecules (substitution of glycine by another amino acid) [25]. The α1 and α2 chains of collagen type I both contain a central triple-helical domain, which is composed of uninterrupted repeats of the Gly-X–Y tripeptide [19]. As glycine is the only small residue to be accommodated inside the helix, triple-helix formation can proceed normally only if a glycine residue is present [26]. In this large cohort of OI, we not only detected many kinds of pathogenic mutations leading to haploinsufficiency and substitution of glycine but also identified 10 biallelic mutational genes that impair multiple aspects of type I collagen, including translation, posttranslational folding, modification, and assembly.

OI is usually overlooked because of misdiagnosis, mild forms in some cases, or remission after puberty [27]. In the current study, the diagnosis of OI (mean age: 10.0 years) was much later than the initial occurrence of bone fracture (mean age: 1.5 years), which was consistent with a previous study [28]. A delayed diagnosis, untimely intervention and management of disease, would lead to a series of adverse consequences, including increased risk of fractures, decreased quality of life, and potentially respiratory and cardiovascular complications [5]. Therefore, it is crucial for doctors to early identify the clinical signs of OI, especially in children with unexplained fractures or a family history of bone fragility disorders [29].

Vitamin D deficiency or insufficiency was common in OI patients [30, 31]. In this study, we found 77.1% (269/349) of OI patients with deficiency or insufficiency of vitamin D. Serum levels of 25OHD decreased with age, which was consistent with previous studies [30–32]. The age-related decline in 25OHD levels may be attributable to OI severity, disease progression, and age-related changes in vitamin D metabolism and neglecting treatment [31, 33].

Recent studies have indicated that genotypes of OI are closely related to phenotypes, but sample sizes were relatively small, and conclusions were not completely consistent [8, 20, 23, 34]. In the present study, we found that haploinsufficiency of collagen type I α chains led to milder skeletal phenotypes than glycine substitution of collagen type I α chains. This finding may explain why patients in this cohort with *COL1A2* mutation had more severe skeletal phenotypes than those with the *COL1A1* mutation. *COL1A1* mutations included 24.2% glycine substitutions and 44.9% haploinsufficiency, whereas the *COL1A2* mutations included 73.0% glycine substitutions. We observed that patients with haploinsufficiency of collagen type I α chains had a lower proportion of wheelchair dependence than those with glycine substitution of collagen type I α chains. Haploinsufficiency produces a half amount of type I collagen with normal structure. Accordingly, patients with haploinsufficiency were more likely to have less fracture and milder skeletal deformities, indicating relatively better mobility than those with glycine substitution in type I collagen.

There are no previous studies comparing OI patients with different ethnicities. We for the first time found that the distribution of biallelic variants differs among countries. *SERPINF1* and *WNT1* were the most common biallelic pathogenic variants in our cohort, which was similar to previous studies of Chinese OI patients [8, 11, 35]. However, this finding was distinct from studies of Western OI patients [20, 21, 23, 36, 37]. Regarding phenotype of OI, we found that the phenotypes were roughly similar between Eastern and Western OI patients and the annual incidence of fractures did not differ statistically significantly among countries. However, the different age, gender, nutritional status, lifestyle of OI patients, and the sample size of different studies made it difficult to complete accurate phenotypic comparisons between different patients' groups. Moreover, it was difficult to exclude the effects of previous treatments, such as bisphosphonates, on phenotypes. It was worth noting that the comparison between Eastern and Western OI patients was based on limited studies and general observations, more in-depth research is worth conducting.

This study obtained an accurate genetic diagnosis of a large sample of OI patients, which is valuable for revealing pathogenic mechanisms and predicting disease prognosis and drug therapy response. Our previous study reported that OI patients with nonautosomal dominant inheritance or with pathogenic mutations leading to collagen structural defects would have relatively poor responses to zoledronic acid treatment [38], indicating that molecular diagnosis is valuable to carry out precision treatment for OI patients. Moreover, gene and cell therapy are currently the most promising treatment prospects for OI [1], and definitive genetic diagnosis may lay the foundation for future gene or cell therapies for these OI patients [39].

Of note, no causative gene mutation was detected in 16.5% of our patients with a clinical diagnosis of OI. Four new OI pathogenic genes (*KDELR2*, *FAM46A*, *MESD* and *CCDC134*) were not included in the NGS panel used, which would reduce the mutation detection rate. In general, whole-genome sequencing (WGS) may be superior for detection of OI pathogenic mutations because it can not only capture noncoding regions of genes but also cover copy number variants, chromosomal rearrangements, and repeat-rich regions [40, 41]. In addition, single-cell RNA sequencing (scRNA-seq) is a powerful tool allowing classification, characterization and distinction of each cell at the transcriptome level [42]. These technologies should be utilized in our cohort.

In this study, we delved into the pathogenic mutations and phenotypic correlation in the largest Asian cohort of OI patients. Data on the phenotype and genotype of OI patients were detailed. All parameters of this study were detected in a single center, which could minimize measurement bias. However, this study had several limitations. This was a retrospective study, and some data were unavailable, especially for patients who visited PUMCH 20 years ago. Additionally, effects of puberty on height and BMD could not be ruled out. Extraskeletal phenotypes have not been fully evaluated. An incomplete panel not covering all known disease-causing genes would affect the accuracy of molecular diagnosis.

In conclusion, detailed genotypes and phenotypes were obtained from the largest cohort of Asian OI patients, enriching the spectrum of OI. A close correlation between genotype and phenotype of OI patients is demonstrated by our results, which is valuable for prenatal diagnosis, differential diagnosis, elucidating pathogenesis mechanism, and predicting prognosis of OI. Differences in the mutation profile of causative genes were found between Eastern and Western OI patients, but the mechanism deserves in-depth study.

### Supplementary Information

Below is the link to the electronic supplementary material.

Supplementary file1 Supplementary Fig 1 Relationships between age and 25OHD levels in OI patients The levels of 25OHD were negatively correlated with age Supplementary Fig 2. Relationships between the position of glycine substitution in collagen type I α chains and phenotypes a. Relationship between the position of glycine substitution in collagen type I α chains and frequency of fracture b. Relationship between the position of glycine substitution in collagen type I α chains and LS BMD Z-score c. Relationship between the position of glycine substitution in collagen type I α chains and FN BMD Z-score d. Relationship between the position of glycine substitution in collagen type I α chains and height Z-score (PPTX 2638 KB)

Supplementary file2 (DOCX 104 KB)

Supplementary file3 (DOCX 14 KB)

## Data Availability

Data are available on reasonable request. The data that support the findings of this study are available from the corresponding author on reasonable request.
