# Supplementary material for: Genotype–phenotype relationship and comparison between eastern and western patients with osteogenesis imperfecta
Source: J Endocrinol Invest. 2023 Jun 4;47(1):67–77. doi: 10.1007/s40618-023-02123-2 (PMC10776744; doi:10.1007/s40618-023-02123-2)
Supplement: Supplementary file 1 — Supplementary file1 Supplementary Fig 1 Relationships between age and 25OHD levels in OI patients The levels of 25OHD were negatively correlated with age Supplementary Fig 2. Relationships between the position of glycine substitution in collagen type I α chains and phenotypes a. Relationship between the position of glycine substitution in collagen type I α chains and frequency of fracture b. Relationship between the position of glycine substitution in collagen type I α chains and LS BMD Z-score c. Relationship between the position of glycine substitution in collagen type I α chains and FN BMD Z-score d. Relationship between the position of glycine substitution in collagen type I α chains and height Z-score (PPTX 2638 KB) [file 40618_2023_2123_MOESM1_ESM.pptx]

## Slide 1
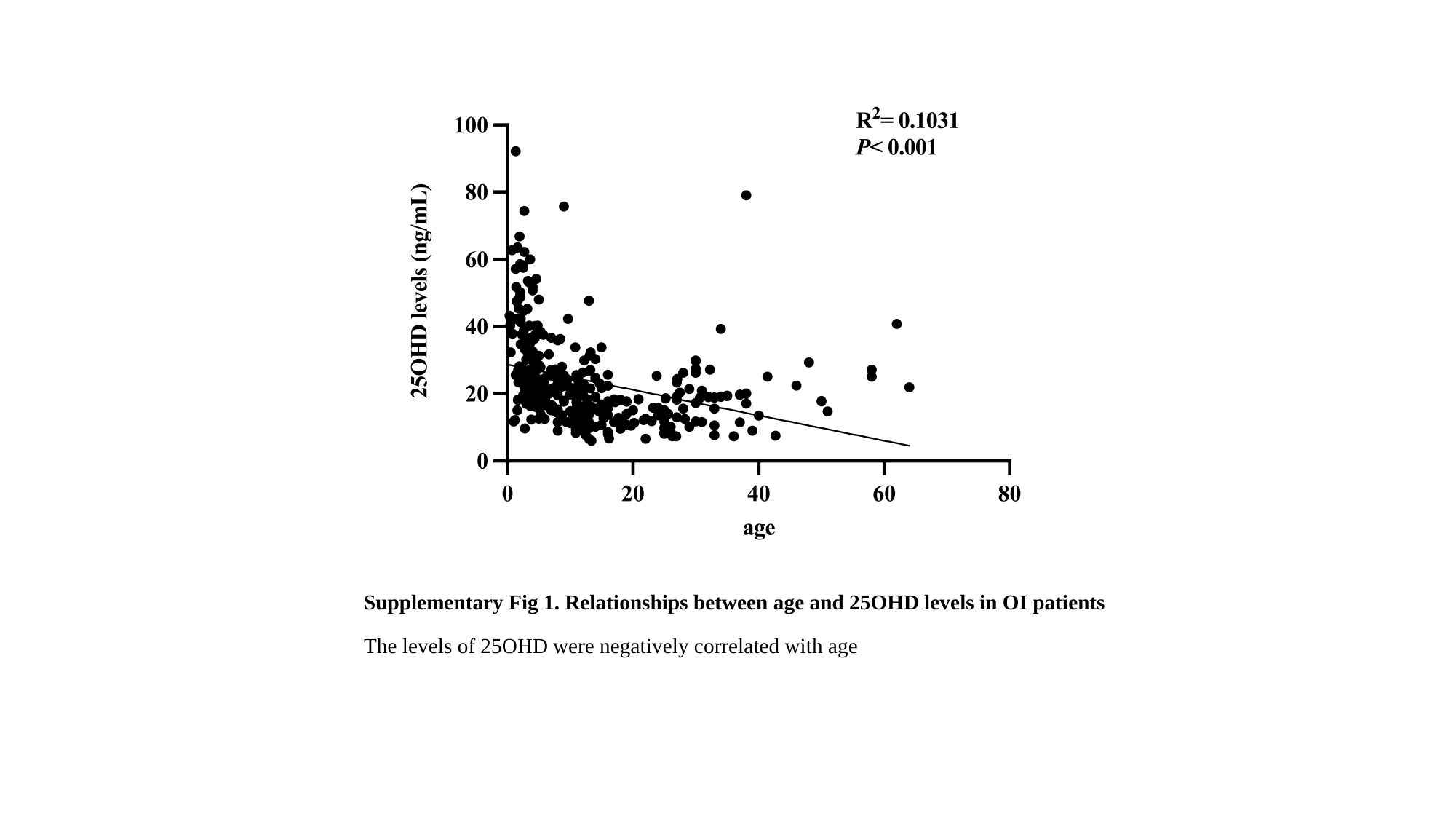

Supplementary Fig 1. Relationships between age and 25OHD levels in OI patients
The levels of 25OHD were negatively correlated with age

## Slide 2
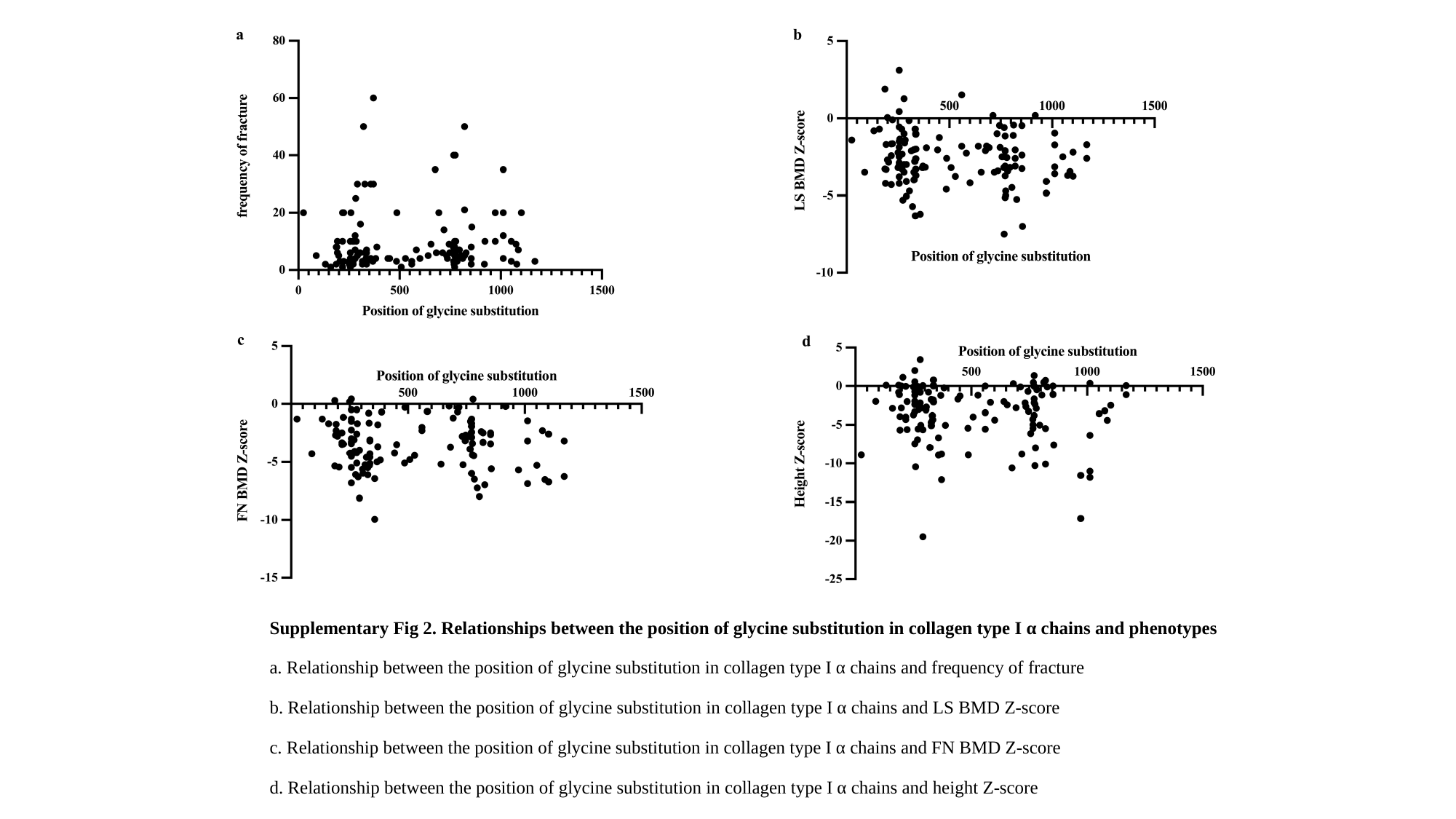

Supplementary Fig 2. Relationships between the position of glycine substitution in collagen type I α chains and phenotypes
a. Relationship between the position of glycine substitution in collagen type I α chains and frequency of fracture
b. Relationship between the position of glycine substitution in collagen type I α chains and LS BMD Z-score
c. Relationship between the position of glycine substitution in collagen type I α chains and FN BMD Z-score
d. Relationship between the position of glycine substitution in collagen type I α chains and height Z-score
