# Supplementary material for: Genotype–phenotype relationship and comparison between eastern and western patients with osteogenesis imperfecta
Source: J Endocrinol Invest. 2023 Jun 4;47(1):67–77. doi: 10.1007/s40618-023-02123-2 (PMC10776744; doi:10.1007/s40618-023-02123-2)
Supplement: Supplementary file 2 — Supplementary file2 (DOCX 104 KB) [file 40618_2023_2123_MOESM2_ESM.docx]

**Supplementary Table 1. Gene mutations identified in 427 probands with osteogenesis imperefcta**

**Supplementary Table 1a. *COL1A1* mutation**

| **No.** | **EXON/INTRON** | **cDNA** | **Protein** | **Type** | **Familial** | **Type OI**  **(Ⅰ–Ⅴ)** |
| --- | --- | --- | --- | --- | --- | --- |
|  | EXON 1 | c.67C>T | p.Gln23* | Nonsense | yes | Ⅰ |
|  | EXON 2 | c.143dupA | p.H48Qfs*2 | Frameshift | / | Ⅰ |
|  | EXON 2 | c.148C>T | p.R50* | Nonsense | yes | Ⅳ |
|  | EXON 2 | c.268G>T | p.Glu90* | Nonsense | yes | Ⅰ |
|  | EXON 2 | c.279delC | p.Val94Serfs*171 | Frameshift | de novo | Ⅰ |
|  | EXON 2 | c.284_285insC | p.Asp97Argfs*72 | Frameshift | / | Ⅰ |
|  | INTRON 2 | c.298+1G>A | / | Splicing | / | Ⅰ |
|  | INTRON2 | c.299-2A>G | / | Splicing | / | Ⅰ |
|  | INTRON 3 | c.334-9A>G | / | Splicing | / | Ⅰ |
|  | INTRON 3 | c.334-9A>G | / | Splicing | / | / |
|  | EXON 4 | c.358C>T | p.Arg120* | Nonsense | / | Ⅰ |
|  | INTRON 4 | c.370-1G>A | / | Splicing | yes | Ⅰ |
|  | EXON 5 | c.371_372insCCCCGCCCTCTCCCCCTGCAGGG | p.Gly127Leufs*146 | Frameshift | yes | Ⅳ |
|  | EXON 5 | c.391C>T | p.Arg131* | Nonsense | yes | Ⅳ |
|  | EXON 5 | c.399G＞T | p. Gly133Val | Missense | / | Ⅳ |
|  | EXON 5 | c.432delC | p.Gly145Aspfs*120 | Frameshift | / | Ⅰ |
|  | EXON 5 | c.441_442insC | p.Gly148Argfs*21 | Frameshift | de novo | Ⅰ |
|  | EXON 5 | c.442G>T | p.Gly148* | Nonsense | / | Ⅰ |
|  | EXON 5 | c.443_444insG | p.Pro149Thrfs*20 | Frameshift | / | Ⅰ |
|  | EXON 7 | c.578dupC | p.Gly194Trpfs*14 | Frameshift | / | Ⅰ |
|  | INTRON 7 | c.588+1G>T | / | Splicing | / | Ⅰ |
|  | INTRON 7 | c.588+4A>T | / | Splicing | / | Ⅰ |
|  | EXON 8 | c.590_599delGTCCCCAAGG | p.Gly197Alafs*65 | Frameshift | yes | Ⅰ |
|  | EXON 8 | c.604C>T | p.Gln202* | Nonsense | yes | Ⅰ |
|  | EXON 8 | c.608G>T | p.Gly25Val | Missense | / | Ⅲ |
|  | INTRON 8 | c.642+5G>A | / | Splicing | / | Ⅰ |
|  | INTRON 8 | c.642+5G>A | / | Splicing | / | / |
|  | EXON 9 | c.643-13_662delCTATCTTTTCTAGGGTCCCATGGGTCCCCGAGG | p.A362fs*? | Frameshift | / | Ⅰ |
|  | EXON 9 | c.652G>A | p.Gly218Ser | Missense | de novo | Ⅰ |
|  | EXON 9 | c.653G>T | p.Gly218Val | Missense | / | Ⅲ |
|  | EXON 9 | c.653G>A | p.Gly218Asp | Missense | / | Ⅲ |
|  | EXON 9 | c.658C>T | p.Arg220* | Nonsense | / | Ⅰ |
|  | EXON 9 | c.658C>T | p.Arg220* | Nonsense | / | Ⅰ |
|  | EXON 9 | c.668dupC | p.Gly224Argfs*9 | Frameshift | / | Ⅰ |
|  | EXON 9 | c.671G>T | p.Gly224Val | Missense | / | Ⅰ |
|  | EXON 9 | c.671G>A | p.Gly224Asp | Missense | / | Ⅲ |
|  | EXON 9 | c.695delA | p.Asp232Valfs*33 | Frameshift | / | Ⅰ |
|  | INTRON 9 | c.697-1G>T | / | Splicing | de novo | Ⅰ |
|  | EXON 10 | c.732_733delTG | p.Gly245Alafs*41 | Frameshift | de novo | Ⅰ |
|  | INTRON 10 | c.750+1G>A | / | Splicing | de novo | Ⅰ |
|  | INTRON 10 | c.750+1G>A | / | Splicing | / | Ⅲ |
|  | INTRON 10 | c.751-2A>C | / | Splicing | de novo | Ⅰ |
|  | INTRON 10 | c.751-2A>C | / | Splicing | / | / |
|  | INTRON 10 | c.751-2A>C | / | Splicing | / | / |
|  | EXON 11 | c.752G>T | p.Gly251Val | Missense | / | Ⅰ |
|  | EXON 11 | c.752G>T | p.Gly251Val | Missense | / | Ⅳ |
|  | EXON 11 | c.769G>A | p.Gly257Arg | Missense | / | Ⅰ |
|  | EXON 11 | c.769G>A | p.Gly257Arg | Missense | de novo | Ⅰ |
|  | EXON 11 | c.769G>A | p.Gly257Arg | Missense | yes | Ⅰ |
|  | EXON 11 | c.769G>A | p.Gly257Arg | Missense | yes | Ⅰ |
|  | EXON 11 | c.769G>A | p.Gly257Arg | Missense | yes | Ⅰ |
|  | EXON 11 | c.769G>A | p.Gly257Arg | Missense | / | Ⅲ |
|  | EXON 11 | c.769G>A | p.Gly257Arg | Missense | / | Ⅰ |
|  | EXON 11 | c.769G>A | p.Gly257Arg | Missense | / | Ⅳ |
|  | EXON 11 | c.769G>A | p.Gly257Arg | Missense | / | Ⅰ |
|  | EXON 11 | c.769G>A | p.Gly257Arg | Missense | / | Ⅰ |
|  | EXON 11 | c.770G>A | p.Gly257Glu | Missense | yes | Ⅳ |
|  | EXON 11 | c.779G>A | p.Gly260Asp | Missense | / | Ⅲ |
|  | EXON 11 | c.801_802delC | p.His267Glnfs*19 | Frameshift | / | Ⅰ |
|  | INTRON 11 | c.802_804+9delAGAGTGAGTCAC | / | Splicing | / | Ⅲ |
|  | EXON 12 | c.806G>T | p.Gly269Val | Missense | / | Ⅲ |
|  | EXON 12 | c.813_814delTG | p.Ser271Argfs*15 | Nonsense | yes | Ⅰ |
|  | EXON 12 | c.823G>A | p.Gly275Ser | Missense | / | Ⅲ |
|  | EXON 14 | c.905delG | p.Gly302Alafs*239 | Frameshift | / | Ⅲ |
|  | EXON 14 | c.913G>A | p.Gly305Ser | Missense | yes | Ⅳ |
|  | EXON 15 | c.959delG | p.Gly320Valfs*221 | Frameshift | yes | Ⅰ |
|  | EXON 15 | c.994G>A | p.Gly332Arg | Missense | / | Ⅰ |
|  | EXON 15 | c.996-998delGCC | p.Pro334Glyfs* | Frameshift | / | Ⅳ |
|  | EXON 15 | c.1001delC | p.Pro334Leufs*207 | Frameshift | yes | Ⅳ |
|  | INTRON 15 | c.1002+5G>A | / | Splicing | / | Ⅲ |
|  | EXON 16 | c.1003G>A | p.Gly335Ser | Missense | yes | Ⅰ |
|  | EXON 17 | c.1060G>T | p.Glu354* | Nonsense | / | Ⅰ |
|  | EXON 17 | c.1077delG | p.Arg361Glufs*180 | Frameshift | / | Ⅰ |
|  | EXON 17 | c.1081C>T | p.Arg361* | Nonsense | yes | Ⅰ |
|  | EXON 17 | c.1081C>T | p.Arg361* | Nonsense | yes | Ⅰ |
|  | EXON 17 | c.1081C>T | p.Arg361* | Nonsense | yes | Ⅰ |
|  | EXON 17 | c.1103G>C | p.Gly368Ala | Missense | / | Ⅳ |
|  | EXON 17 | c.1111G>A | p.Gly371Ser | Missense | / | Ⅲ |
|  | EXON 17 | c.1111G>A | p.Gly371Ser | Missense | / | Ⅲ |
|  | EXON 17 | c.1127delC | p.Pro376Leufs*165 | Frameshift | / | Ⅳ |
|  | INTRON 17 | c.1155+1G>C | / | Splicing | / | Ⅳ |
|  | INTRON 17 | c.1155+1G>C | / | Splicing | de novo | Ⅰ |
|  | INTRON 17 | c.1155+1G>C | / | Splicing | de novo | Ⅰ |
|  | INTRON 17 | c.1155+1G>C | / | Splicing | / | Ⅰ |
|  | INTRON 17 | c.1155+1G>C | / | Splicing | yes | Ⅳ |
|  | INTRON 18 | c.1200+1G>T | / | Splicing | yes | Ⅰ |
|  | EXON 19 | c.1243C>T | p.Arg415* | Nonsense | yes | Ⅰ |
|  | EXON 19 | c.1243C>T | p.Arg415* | Nonsense | / | Ⅰ |
|  | EXON 19 | c.1243C>T | p.Arg415* | Nonsense | / | Ⅰ |
|  | INTRON 19 | c.1299+1G>A | / | Splicing | yes | Ⅰ |
|  | INTRON 19 | c.1299+1G>A | / | Splicing | yes | Ⅰ |
|  | INTRON 19 | c.1299+1G>A | / | Splicing | yes | Ⅰ |
|  | INTRON 19 | c.1299+1G>A | / | Splicing | / | Ⅰ |
|  | INTRON 19 | c.1299+1G>A | / | Splicing | de novo | Ⅰ |
|  | INTRON 19 | c.1300-1G>A | / | Splicing | / | Ⅰ |
|  | INTRON 20 | c.1354-12G>A | / | Splicing | de novo | Ⅳ |
|  | EXON 21 | c.1373delG | p.Gly458Aspfs*83 | Frameshift | / | Ⅰ |
|  | EXON 21 | c.1386delT | p.Ala463Leufs*78 | Frameshift | yes | Ⅰ |
|  | EXON 21 | c.1408G>T | p.Gly470* | Nonsense | yes | Ⅳ |
|  | EXON 21 | c.1414C>T | p.Arg472* | Nonsense | / | Ⅰ |
|  | EXON 21 | c.1414C>T | p.Arg472* | Nonsense | yes | Ⅰ |
|  | EXON 21 | c.1414C>T | p.Arg472* | Nonsense | / | Ⅰ |
|  | EXON 21 | c.1451delC | p.Pro484Leufs*57 | Frameshift | de novo | Ⅰ |
|  | EXON 21 | c.1454G>C | p.Gly485Ala | Missense | / | Ⅲ |
|  | INTRON 21 | c.1461+1G>A | / | Splicing | / | Ⅰ |
|  | INTRON 21 | c.1515+3A>G | / | Splicing | yes | Ⅲ |
|  | INTRON 22 | c.1516-5A>G |  | Splicing | / | Ⅳ |
|  | EXON 23 | c.1524delT | p.Gly509Valfs*32 | Frameshift | yes | Ⅰ |
|  | EXON 23 | c.1531_1534dupCGTG | p.Gly512Alafs*15 | Frameshift | / | Ⅰ |
|  | EXON 23 | c.1591G>A | p.Glu531Lys | Missense | / | Ⅳ |
|  | EXON 24 | c.1661delG | p.Gly554Alafs*26 | Frameshift | / | Ⅰ |
|  | EXON 24 | c.1667delC | p.Pro556Leufs*24 | Frameshift | / | Ⅰ |
|  | EXON 24 | c.1667dupC | p.Gly557Trpfs*30 | Frameshift | / | Ⅰ |
|  | EXON 24 | c.1668delT | p.Gly557Valfs*23 | Frameshift | yes | Ⅲ |
|  | EXON 25 | c.1678G>A | p.Gly560Ser | Missense | yes | Ⅲ |
|  | EXON 25 | c.1678G>A | p.Gly560Ser | Missense | yes | Ⅳ |
|  | EXON 25 | c.1678G>A | p.Gly560Ser | Missense | yes | Ⅳ |
|  | EXON 26 | c.1792C>T | p.Arg598* | Nonsense | / | Ⅰ |
|  | EXON 26 | c.1792C>T | p.Arg598* | Nonsense | / | Ⅰ |
|  | INTRON 26 | c.1821+1G>A | / | Splicing | yes | Ⅰ |
|  | INTRON 26 | c.1821+1G>A | / | Splicing | yes | Ⅰ |
|  | INTRON 26 | c.1821+1G>A | / | Splicing | yes | Ⅰ |
|  | INTRON 26 | c.1821+1G>A | / | Splicing | / | Ⅰ |
|  | INTRON 26 | c.1822-2A>T | / | Splicing | / | Ⅳ |
|  | INTRON 26 | c.1822-2delA | / | Splicing | / | Ⅰ |
|  | EXON 28 | c.1922delG | p.Gly641Aspfs*125 | Frameshift | yes | Ⅰ |
|  | EXON 28 | c.1922G>C | p.Gly641Ala | Missense | / | Ⅳ |
|  | INTRON 29 | c.1983+1G>A | / | Splicing | / | / |
|  | EXON 30 | c.1985delG | p.Gly662Valfs*104 | Frameshift | / | Ⅰ |
|  | EXON 30 | c.2010delT | p.Gly671Alafs*95 | Frameshift | / | Ⅲ |
|  | EXON 30 | c.2010delT | p.Gly671Alafs*95 | Frameshift | / | Ⅰ |
|  | INTRON 30 | c.2028+4_2028+5delAG | / | Splicing | / | Ⅰ |
|  | INTRON 30 | c.2028+1G>A | / | Splicing | / | Ⅰ |
|  | EXON 31 | c.2037_2038delAG | p.Gly680Phefs*29 | Frameshift | / | Ⅰ |
|  | EXON 31 | c.2073delT | p.Gly692Valfsx*74 | Frameshift | / | Ⅰ |
|  | EXON 31 | c.2089C>T | p.Arg697* | Nonsense | yes | Ⅰ |
|  | EXON 32 | c.2137G>A | p.Gly713Ser | Missense | / | Ⅰ |
|  | EXON 32 | c.2155G>A | p.Gly719Ser | Missense | / | Ⅲ |
|  | EXON 32 | c.2216C>T | p.Pro739Leu | Missense | / | / |
|  | INTRON 32 | c.2235_2235+1delAG | / | Splicing | / | Ⅰ |
|  | EXON 33 | c.2243delC | p.Ala748Valfs*18 | Frameshift | / | Ⅳ |
|  | EXON 33 | c.2250delC | p.Gly752Valfs*14 | Frameshift | / | Ⅰ |
|  | EXON 33 | c.2280T>A | p.Asp760Glu | Missense | / | Ⅰ |
|  | EXON 33 | c.2285delT | p.Val762Alafs*4 | Frameshift | / | Ⅰ |
|  | EXON 33 | c.2299G>A | p.Gly767Ser | Missense | / | Ⅲ |
|  | EXON 33 | c.2299G>A | p.Gly767Ser | Missense | / | Ⅲ |
|  | EXON 33 | c.2334delT | p.Gly779Valfs*329 | Frameshift | de novo | Ⅰ |
|  | EXON 34 | c.2362G>A | p.Gly788Ser | Missense | / | Ⅳ |
|  | EXON 35 | c.2434G>A | p.Gly812Ser | Missense | / | Ⅳ |
|  | EXON 35 | c.2450_2451insC | p.Gly818Trpfs*3 | Frameshift | / | Ⅰ |
|  | EXON 36 | c.2450delC | p.Pro817Leufs*291 | Frameshift | / | Ⅰ |
|  | EXON 36 | c.2461G>A | p.Gly821Ser | Missense | yes | Ⅲ |
|  | EXON 36 | c.2461G>A | p.Gly821Ser | Missense | / | Ⅲ |
|  | EXON 36 | c.2461G>A | p.Gly821Ser | Missense | / | Ⅳ |
|  | EXON 36 | c.2461G>A | p.Gly821Ser | Missense | / | Ⅳ |
|  | EXON 36 | c.2482G>A | p.Glu828Lys | Missense | / | Ⅰ |
|  | EXON 36 | c.2522delC | p.Pro841Leufs*267 | Frameshift | / | Ⅰ |
|  | EXON 36 | c.2523delT | p.Gly842Alafs*266 | Frameshift | yes | Ⅰ |
|  | EXON 36 | c.2549delC | p.Pro850Leufs*258 | Frameshift | / | Ⅰ |
|  | EXON 37 | c.2560G>A | p.Gly854Ser | Missense | yes | Ⅰ |
|  | EXON 37 | c.2560G>A | p.Gly854Ser | Missense | yes | Ⅰ |
|  | EXON 37 | c.2560G>A | p.Gly854Ser | Missense | / | Ⅰ |
|  | EXON 37 | c.2563_2565dupAAT | p.Asn855_Val856insAsn | Frameshift | de novo | Ⅳ |
|  | EXON 37 | c.2569G>T | p.Gly857Cys | Missense | / | Ⅲ |
|  | EXON 38 | c.2588delG | p.Gly863Valfs*245 | Frameshift | yes | Ⅰ |
|  | EXON 38 | c.2644C>T | p.Arg882* | Nonsense | yes | Ⅳ |
|  | EXON 38 | c.2644C>T | p.Arg882* | Nonsense | yes | Ⅰ |
|  | EXON 38 | c.2644C>T | p.Arg882* | Nonsense | de novo | Ⅰ |
|  | EXON 38 | c.2644C>T | p.Arg882* | Nonsense | de novo | Ⅰ |
|  | INTRON 38 | c.2667+1G>A | / | Splicing | de novo | Ⅳ |
|  | EXON 39 | c.2739delTinsCA | p.Gly914Argfs*8 | Frameshift | / | Ⅰ |
|  | EXON 39 | c.2784delT | p.Gly929Alafs*179 | Frameshift | yes | Ⅰ |
|  | INTRON 39 | c.2829+4delA | / | Splicing | / | Ⅰ |
|  | EXON 40 | c.2858delG | p.Gly953Valfs*155 | Frameshift | / | Ⅰ |
|  | EXON 41 | c.3008delC | p.Pro1003Leufs*15 | Frameshift | / | Ⅰ |
|  | EXON 41 | c.3026delC | p.Pro1009Leufs*99 | Frameshift | yes | Ⅰ |
|  | EXON 41 | c.3040C>T | p.Arg1014Cys | Missense | yes | Ⅳ |
|  | EXON 41 | c.3040C>T | p.Arg1014Cys | Missense | / | Ⅳ |
|  | INTRON 41 | c.3045+1G>A | / | Splicing | de novo | Ⅳ |
|  | EXON 42 | c.3063_3064delAG | p.Gly1022Phefs*13 | Frameshift | / | Ⅳ |
|  | EXON 42 | c.3076C>T | p.Arg1026* | Nonsense | yes | Ⅰ |
|  | EXON 42 | c.3076C>T | p.Arg1026* | Nonsense | / | Ⅰ |
|  | EXON 42 | c.3076C>T | p.Arg1026* | Nonsense | / | Ⅲ |
|  | EXON 42 | c.3076C>T | p.Arg1026* | Nonsense | / | Ⅳ |
|  | EXON 42 | c.3076C>T | p.Arg1026* | Nonsense | de novo | Ⅰ |
|  | EXON 42 | c.3076C>T | p.Arg1026* | Nonsense | yes | Ⅳ |
|  | EXON 43 | c.3135delT | p.Gly1046Valfs*62 | Frameshift | / | Ⅳ |
|  | EXON 43 | c.3155G>C | p.Gly1052Ala | Missense | / | Ⅳ |
|  | EXON 43 | c.3155G>C | p.Gly1052Ala | Missense | / | Ⅲ |
|  | EXON 44 | c.3209_3218delGTCCTGCTGG | p.Gly1070Valfs*35 | Frameshift | yes | Ⅰ |
|  | EXON 44 | c.3226G>A | p.Gly1076Ser | Missense | / | Ⅳ |
|  | EXON 44 | c.3235G>A | p.Gly1079Ser | Missense | yes | Ⅳ |
|  | EXON 44 | c.3258delC | p.Ala1087Profs*21 | Frameshift | yes | Ⅳ |
|  | EXON 44 | c.3260C>A | p.Ala1087Asp | Missense | / | Ⅰ |
|  | EXON 44 | c.3260C>A | p.Ala1087Asp | Missense | yes | Ⅰ |
|  | INTRON 44 | c.3262-1G>A | / | Splicing | / | Ⅰ |
|  | INTRON 44 | c.3262-1G>T | / | Splicing | de novo | Ⅰ |
|  | EXON 45 | c.3360del | c.Gly1121Alafs*118 | Frameshift | de novo | Ⅰ |
|  | INTRON 45 | c.3369+1G>A | / | Splicing | / | Ⅳ |
|  | INTRON 45 | c.3369+1G>C | / | Splicing | yes | Ⅰ |
|  | EXON 46 | c.3421C>T | p.Arg1141* | Nonsense | yes | Ⅰ |
|  | EXON 46 | c.3421C>T | p.Arg1141* | Nonsense | de novo | Ⅰ |
|  | EXON 47 | c.3466_3472delAACGGTC | p.Asn1156Serfs*81 | Frameshift | yes | Ⅳ |
|  | EXON 47 | c.3505G>A | p.Gly1169Ser | Missense | / | Ⅳ |
|  | EXON 47 | c.3505G>A | p.Gly1169Ser | Missense | de novo | Ⅰ |
|  | EXON 47 | c.3521C>T | p.Ala1174Val | Missense | yes | Ⅳ |
|  | INTRON 47 | c.3531+3A>T | / | Splicing | / | Ⅳ |
|  | INTRON 47 | c.3531+3A>T | / | Splicing | / | Ⅰ |
|  | INTRON 47 | c.3531+1G>T | / | Splicing | / | Ⅳ |
|  | INTRON 47 | c.3531+5G>A | / | Splicing | / | Ⅲ |
|  | INTRON 47 | c.3531+1G>A | / | Splicing | / | Ⅰ |
|  | EXON 48 | c.3540_3541insC | p.Gly1181fs*39 | Frameshift | / | Ⅰ |
|  | EXON 48 | c.3566delC | p.Pro1189Leufs*50 | Frameshift | / | Ⅰ |
|  | EXON 48 | c.3627delT | p.His1210Thrfs*29 | Frameshift | yes | Ⅰ |
|  | EXON 48 | c.3633dupT | p.Gly1212Trpfs*8 | Frameshift | / | Ⅰ |
|  | EXON 48 | c.3652delG | p.Ala1218Leufs*21 | Frameshift | de novo | Ⅰ |
|  | EXON 48 | c.3766G>A | p.Ala1256Thr | Missense | yes | Ⅲ |
|  | EXON 48 | c.3766G>A | p.Ala1256Thr | Missense | de novo | Ⅰ |
|  | INTRON 48 | c.3814+1G>T | / | Splicing | yes | Ⅰ |
|  | EXON 49 | c.3823T>A | p.Trp1275Arg | Missense | yes | Ⅰ |
|  | EXON 49 | c.3857C>A | p.Gly772Ser | Missense | yes | Ⅳ |
|  | EXON 49 | c.3857C>A | p.Ala1286Asp | Missense | yes | Ⅳ |
|  | EXON 49 | c.3893C>A | p.Thr1298Asn | Missense | / | Ⅰ |
|  | EXON 49 | c.3898delG | p.Val1300Cysfs*31 | Frameshift | / | Ⅳ |
|  | EXON 49 | c.3935G>A | p.Trp1312* | Nonsense | / | Ⅰ |
|  | INTRON 49 | c.4005+1G>A | / | Splicing | / | Ⅰ |
|  | EXON 51 | c.4316T>C | p.Ile1439Thr | Missense | yes | Ⅲ |
|  | EXON 51 | c.4316T>C | p.Ile1439Thr | Missense | yes | Ⅰ |
|  | EXON 51 | c.4328C>T | p.Ala1443Val | Missense | / | Ⅳ |
|  | EXON 51 | c.4328C>T | p.Ala1443Val | Missense | de novo | Ⅳ |
|  | EXON 51 | c.4381_4382delGT | p.Val1461Leufs*89 | Frameshift | / | Ⅰ |
|  | EXON 1-51 | exon1_51  deletion | / | Multi-exon deletion | / | Ⅰ |
|  | EXON 1-51 | exon1_51  deletion | / | Multi-exon deletion | / | Ⅰ |

**Supplementary Table 1b. *COL1A2* mutation**

| **No.** | **EXON/INTRON** | **cDNA** | **Protein** | **Type** | **Familial** | **Type OI**  **(Ⅰ–Ⅴ)** |
| --- | --- | --- | --- | --- | --- | --- |
|  | EXON 10 | c.478G>A | p.Gly160Arg | Missense | yes | Ⅰ |
|  | EXON 12 | c.560G>A | p.Gly187Glu | Missense | yes | Ⅰ |
|  | EXON 12 | c.560G>A | p.Gly187Glu | Missense | / | Ⅰ |
|  | EXON 12 | c.568G>C | p.Gly190Arg | Missense | / | Ⅰ |
|  | EXON 12 | c.578G>T | p.Gly193Val | Missense | / | Ⅳ |
|  | EXON 12 | c.578G>A | p.Gly193Asp | Missense | / | Ⅳ |
|  | EXON 13 | c.596G>T | p.Gly199Val | Missense | yes | Ⅳ |
|  | EXON 13 | c.596G>A | p.Gly199Asp | Missense | yes | Ⅰ |
|  | EXON 13 | c.614G>A | p.Gly205Asp | Missense | / | Ⅰ |
|  | INTRON 14 | c.693+1G>A | / | Splicing | yes | Ⅳ |
|  | INTRON 14 | c.693+2T>A | / | Splicing | / | Ⅳ |
|  | INTRON 14 | c.694-1G>C | / | Splicing | / | Ⅳ |
|  | EXON 15 | c.757C>T | p.Arg253* | Nonsense | de novo | Ⅰ |
|  | INTRON 16 | c.792+3A>T | / | Splicing | yes | Ⅰ |
|  | EXON 17 | c.811G>C | p.Gly271Arg | Missense | yes | Ⅲ |
|  | EXON 17 | c.812G>A | p.Gly271Asp | Missense | / | Ⅰ |
|  | EXON 17 | c.838G>A | p.Gly280Ser | Missense | yes | Ⅳ |
|  | EXON 17 | c.838G>A | p.Gly280Ser | Missense | / | Ⅰ |
|  | EXON 17 | c.838G>A | p.Gly280Ser | Missense | / | Ⅳ |
|  | EXON 17 | c.838G>A | p.Gly280Ser | Missense | yes | Ⅰ |
|  | EXON 17 | c.847G>A | p.Gly283Ser | Missense | / | Ⅲ |
|  | EXON 17 | c.856G>A | p.Gly286Ser | Missense | yes | Ⅲ |
|  | EXON 17 | c.874G>C | p.Gly292Arg | Missense | yes | Ⅰ |
|  | EXON 17 | c.875G>A | p.Gly292Asp | Missense | yes | Ⅳ |
|  | EXON 17 | c.875G>A | p.Gly292Asp | Missense | yes | Ⅲ |
|  | EXON 18 | c.910G>C | p.Gly304Arg | Missense | / | / |
|  | EXON 18 | c.920G>A | p.Gly307Asp | Missense | / | Ⅳ |
|  | EXON 19 | c.946G>A | p.Gly316Ser | Missense | yes | Ⅳ |
|  | EXON 19 | c.964G>A | p.Gly322Ser | Missense | yes | Ⅲ |
|  | EXON 19 | c.946G>A | p.Gly316Ser | Missense | yes | Ⅳ |
|  | EXON 19 | c.982G>A | p.Gly328Ser | Missense | / | Ⅳ |
|  | EXON 19 | c.982G>A | p.Gly328Ser | Missense | / | Ⅲ |
|  | EXON 19 | c.982G>A | p.Gly328Ser | Missense | / | Ⅳ |
|  | EXON 19 | c.1000G>A | p.Gly334Ser | Missense | / | Ⅳ |
|  | EXON 19 | c.1009G>A | p.Gly337Ser | Missense | yes | Ⅲ |
|  | EXON 19 | c.1009G>A | p.Gly337Ser | Missense | / | Ⅰ |
|  | EXON 19 | c.1009G>A | p.Gly337Ser | Missense | / | Ⅰ |
|  | EXON 19 | c.1009G>A | p.Gly337Ser | Missense | yes | Ⅰ |
|  | EXON 19 | c.1009G>A | p.Gly337Ser | Missense | yes | Ⅳ |
|  | EXON 19 | c.1009G>A | p.Gly337Ser | Missense | yes | Ⅳ |
|  | EXON 19 | c.1009G>A | p.Gly337Ser | Missense | / | / |
|  | EXON 20 | c.1072G>A | p.Gly358Ser | Missense | / | Ⅲ |
|  | EXON 20 | c.1072G>A | p.Gly358Ser | Missense | / | Ⅲ |
|  | EXON 21 | c.1144G>A | p.Gly382Ser | Missense | yes | Ⅳ |
|  | EXON 21 | c.1162G>A | p.Gly388Arg | Missense | / | Ⅳ |
|  | INTRON 21 | c.1197+5G>A | / | Splicing | yes | Ⅲ |
|  | INTRON 21 | c.1197+5G>A | / | Splicing | / | Ⅳ |
|  | INTRON 21 | c.1197+5G>A | / | Splicing | / | Ⅰ |
|  | EXON 23 | c.1325G>A | p.Gly442Glu | Missense | / | Ⅳ |
|  | EXON 24 | c.1352G>C | p.Gly451Ala | Missense | / | Ⅰ |
|  | EXON 24 | c.1384G>T | p.Ala462Ser | Missense | / | Ⅳ |
|  | EXON 25 | c.1459G>A | p.Gly487Arg | Missense | / | Ⅲ |
|  | EXON 26 | c.1523G>T | p.Gly508Val | Missense | / | Ⅳ |
|  | EXON 27 | c.1586G>A | p.Gly529Asp | Missense | / | Ⅰ |
|  | EXON 30 | c.1748G>C | p.Gly583Ala | Missense | yes | Ⅳ |
|  | EXON 31 | c.1801G>A | p.Gly601Ser | Missense | yes | Ⅳ |
|  | EXON 32 | c.1963G>A | p.Gly655Arg | Missense | / | Ⅰ |
|  | EXON 34 | c.2027G>T | p.Gly676Val | Missense | / | Ⅲ |
|  | EXON 34 | c.2038_2055dupGCTGTAGGTGCCCCTGGT | p.Ala680_Gly685dup | Frameshift | de novo | Ⅲ |
|  | EXON 34 | c.2044G>C | p.Gly682Arg | Missense | yes | Ⅰ |
|  | EXON 35 | c.2081G>A | p.Gly694Asp | Missense | yes | Ⅰ |
|  | EXON 35 | c.2121_2122insGCTGGTCCT | p.Pro707_Arg708ins  AlaGlyPro | Frameshift | yes | Ⅳ |
|  | INTRON 35 | c.2133+6T>A | / | Splicing | yes | Ⅰ |
|  | INTRON 35 | c.2133+6T>A | / | Splicing | yes | Ⅰ |
|  | EXON 37 | c.2197G>T | p.Gly733Cys | Missense | / | Ⅰ |
|  | EXON 37 | c.2206G>C | p.Gly736Arg | Missense | / | Ⅳ |
|  | EXON 37 | c.2233G>A | p.Gly745Arg | Missense | yes | Ⅰ |
|  | EXON 37 | c.2242G>A | p.Gly748Ser | Missense | / | Ⅳ |
|  | EXON 37 | c.2269G>A | p.Gly757Ser | Missense | / | Ⅳ |
|  | EXON 38 | c.2296G>A | p.Gly766Ser | Missense | / | Ⅳ |
|  | EXON 38 | c.2305G>T | p.Gly769Cys | Missense | / | Ⅰ |
|  | EXON 38 | c.2314G>A | p.Gly772Ser | Missense | yes | Ⅳ |
|  | EXON 38 | c.2314G>A | p.Gly772Ser | Missense | yes | Ⅲ |
|  | EXON 38 | c.2314G>A | p.Gly772Ser | Missense | de novo | Ⅳ |
|  | EXON 38 | c.2314G>A | p.Gly772Ser | Missense | yes | Ⅰ |
|  | EXON 38 | c.2314G>A | p.Gly772Ser | Missense | / | Ⅳ |
|  | EXON 38 | c.2323G>A | p.Gly775Arg | Missense | / | Ⅳ |
|  | EXON 38 | c.2324G>A | p.Gly775Glu | Missense | / | Ⅲ |
|  | EXON 38 | c.2330G>A | p.Arg777His | Missense | / | Ⅲ |
|  | EXON 38 | c.2333G>A | p.Gly778Asp | Missense | yes | Ⅲ |
|  | EXON 38 | c.2341G>A | p.Gly781Ser | Missense | / | Ⅳ |
|  | EXON 39 | c.2350G>A | p.Gly784Ser | Missense | / | Ⅳ |
|  | EXON 39 | c.2387G>C | p.Gly796Ala | Missense | / | Ⅲ |
|  | EXON 40 | c.2413G>C | p.Gly805Arg | Missense | yes | Ⅰ |
|  | EXON 40 | c.2440G>A | p.Gly814Arg | Missense | yes | Ⅰ |
|  | EXON 40 | c.2456G>A | p.Arg819His | Missense | yes | Ⅳ |
|  | EXON 40 | c.2456G>A | p.Arg819His | Missense | / | Ⅰ |
|  | EXON 40 | c.2485G>A | p.Gly829Ser | Missense | / | Ⅰ |
|  | INTRON 40 | c.2565+1G>A | / | Splicing | / | Ⅲ |
|  | INTRON 40 | c.2565+1G>A | / | Splicing | / | Ⅳ |
|  | EXON 42 | c.2756G>A | p.Gly919Asp | Missense | de novo | Ⅰ |
|  | EXON 42 | c.2764G>C | p.Gly922Arg | Missense | / | Ⅲ |
|  | INTRON 43 | c.2835+1G>A | / | Splicing | yes | Ⅳ |
|  | INTRON 43 | c.2835+1G>A | / | Splicing | / | Ⅳ |
|  | EXON 44 | c.2918G>A | p.Gly973Asp | Missense | / | Ⅲ |
|  | EXON 44 | c.2918G>T | p.Gly973Val | Missense | / | Ⅲ |
|  | EXON 46 | c.3034G>A | p.Gly1012Ser | Missense | / | Ⅳ |
|  | EXON 46 | c.3034G>A | p.Gly1012Ser | Missense | / | Ⅳ |
|  | EXON 46 | c.3034G>A | p.Gly1012Ser | Missense | / | Ⅲ |
|  | EXON 46 | c.3034G>A | p.Gly1012Ser | Missense | / | Ⅲ |
|  | EXON 48 | c.3202A>G | p.Thr1068Ala | Missense | / | Ⅰ |
|  | EXON 48 | c.3259G>C | p.Gly1087Arg | Missense | yes | Ⅲ |
|  | EXON 49 | c.3305G>C | p.Gly1102Ala | Missense | / | Ⅳ |
|  | EXON 49 | c.3305G>C | p.Gly1102Ala | Missense | / | Ⅲ |
|  | EXON 49 | c.3313G>A | p.Gly1105Ser | Missense | / | / |
|  | EXON 49 | c.3350A>G | p.Tyr1117Cys | Missense | / | Ⅰ |
|  | EXON 51 | c.3733G>A | p.Val1245Met | Missense | yes | Ⅰ |
|  | EXON 51 | c.3814T>C | p.Cys1272Arg | Missense | / | Ⅳ |
|  | EXON 51 | c.3816C>G | p.Cys1272Trp | Missense | de novo | Ⅳ |
|  | EXON51 | c.3863delA | p.Lys1288Argfs*27 | Frameshift | / | Ⅰ |
|  | EXON 52 | c.4045dupA | p.Ile1349Asnfs*5 | Frameshift | / | Ⅳ |
|  | EXON 1-52 | LOH | / | Deletion | / | Ⅲ |
|  | EXON 7-12 | LOH | / | Deletion | / | Ⅳ |
|  | / | / | / | Chromosome  translocation | / | Ⅳ |

**Supplementary Table 1c. *IFITM5* mutation**

| **No.** | **EXON/INTRON** | **cDNA** | **Protein** | **Type** | **Familial** | **Type OI**  **(Ⅰ–Ⅴ)** |
| --- | --- | --- | --- | --- | --- | --- |
|  | EXON 1/5'UTR | c.-14C>T | / | Missense | de novo | Ⅴ |
|  | EXON 1/5'UTR | c.-14C>T | / | Missense | yes | Ⅴ |
|  | EXON 1/5'UTR | c.-14C>T | / | Missense | de novo | Ⅴ |
|  | EXON 1/5'UTR | c.-14C>T | / | Missense | / | Ⅴ |
|  | EXON 1/5'UTR | c.-14C>T | / | Missense | yes | Ⅴ |
|  | EXON 1/5'UTR | c.-14C>T | / | Missense | / | Ⅴ |
|  | EXON 1/5'UTR | c.-14C>T | / | Missense | yes | Ⅴ |
|  | EXON 1/5'UTR | c.-14C>T | / | Missense | de novo | Ⅴ |
|  | EXON 1/5'UTR | c.119C>T | p.Ser40Leu | Missense | / | Ⅴ |
|  | EXON 1/5'UTR | c.-14C>T | / | Missense | / | Ⅴ |
|  | EXON 1/5'UTR | c.-14C>T | / | Missense | / | Ⅴ |
|  | EXON 1/5'UTR | c.-14C>T | / | Missense | yes | Ⅴ |
|  | EXON 1/5'UTR | c.-14C>T | / | Missense | / | Ⅴ |
|  | EXON 1/5'UTR | c.-14C>T | / | Missense | / | Ⅴ |
|  | EXON 1/5'UTR | c.-14C>T | / | Missense | / | Ⅴ |
|  | EXON 1/5'UTR | c.-14C>T | / | Missense | / | Ⅴ |
|  | EXON 1/5'UTR | c.-14C>T | / | Missense | / | Ⅴ |
|  | EXON 1/5'UTR | c.-14C>T | / | Missense | / | Ⅴ |
|  | EXON 1/5'UTR | c.-14C>T | / | Missense | de novo | Ⅴ |
|  | EXON 1/5'UTR | c.-14C>T | / | Missense | de novo | Ⅴ |
|  | EXON 1/5'UTR | c.-14C>T | / | Missense | / | Ⅴ |

**Supplementary Table 1d. *P4HB* mutation**

| **No.** | **EXON/INTRON** | **cDNA** | **Protein** | **Type** | **Familial** | **Type OI**  **(Ⅰ–Ⅴ)** |
| --- | --- | --- | --- | --- | --- | --- |
|  | EXON 5 | c.692A>C | p.His231Pro | Missense | yes | Ⅰ |

**Supplementary Table 1e. *PLS3* mutation**

| **No.** | **EXON/INTRON** | **cDNA** | **Protein** | **Type** | **Familial** | **Type OI**  **(Ⅰ–Ⅴ)** |
| --- | --- | --- | --- | --- | --- | --- |
|  | EXON 10 | c.1106_1107insGAAA | p.Phe369Leufs*5 | Frameshift | / | Ⅳ |
|  | EXON 10 | c.1106_1108insGAAA | p.Phe369Leufs*6 | Frameshift | / | Ⅰ |
|  | INTRON 2 | c.73+1_73+4delGTGA | / | Frameshift | yes | Ⅰ |
|  | INTRON 2 | c.73+1_73+4delGTGA | / | Frameshift | / | Ⅰ |
|  | INTRON 2 | c.73+1_73+4delGTGA | / | Frameshift | Yes | / |
|  | EXON 9–15 | / | / | Gross deletion | / | Ⅳ |
|  | EXON 14 | c.1566_1582dupCATCATTGT | p.Val528Alafs*4 | Frameshift | / | Ⅰ |

**Supplementary Table 1f. Biallelic variants**

| **No.** | **Gene** | **EXON/INTRON** | **cDNA** | **Protein** | **Type** | **Type OI**  **(Ⅰ–Ⅴ)** |
| --- | --- | --- | --- | --- | --- | --- |
|  | *SERPINF1* | EXON 3/  EXON3–8 | c.271_279dupGCCCTCTCG/  EXON 3–8Edel | p.Ala91_Ser93dup | Frameshift/  Deletion | Ⅳ |
|  | *SERPINF1* | EXON 3/  EXON 3 | c.184G>A/c.167C>G | p.Gly62Ser/  p.Ala56Gly | Missense | Ⅲ |
|  | *SERPINF1* | INTRON 3/  EXON 5 | c.283+1G>T/  c.498_499delCA | p.Arg167Serfs*35 | Splicing/  Frameshift | Ⅳ |
|  | *SERPINF1* | EXON 7 | c.907C>T | p.Arg303* | Nonsense | Ⅰ |
|  | *SERPINF1* | EXON 2–4/  EXON 7 | EX2-4del/c.907C>T | / | Frameshift/  Missense | Ⅳ |
|  | *SERPINF1* | EXON 4 | c.397C>T | p.Gln133X | Nonsense | / |
|  | *SERPINF1* | EXON 4 | c.397C>T | p.Gln133X | Nonsense | / |
|  | *SERPINF1* | INTRON 5 | c.786+1G>A | / | Splicing | Ⅲ |
|  | *SERPINF1* | EXON 7/  EXON 7 | c.973G>T/c.826T>A | p.Val325Phe/  p.Phe276Ile | Missense | Ⅰ |
|  | *SERPINF1* | EXON 7/  EXON 5 | c.839dup/  c.621_623del | p.Lys281Glufs*20/  p.Leu208del | Frameshift | Ⅲ |
|  | *SERPINF1* | EXON 8 | c.1202_1203delCA | p.Thr401ArgfsX | Frameshift | Ⅲ |
|  | *SERPINH1* | EXON 2/  EXON 5 | c.149T>G/c.1214G>A | p.Leu50Arg/  p.Arg405His | Missense | Ⅳ |
|  | *SERPINH1* | EXON 2 | c.188C>T | p.Ala63Val | Missense | Ⅳ |
|  | *SERPINH1* | EXON 2 | c.188C>T | p.Ala63Val | Missense | Ⅰ |
|  | *FKBP10* | EXON 2/  EXON 7 | c.343C>T/c.1085delC | p.Arg115*/  p.Ala362fs*? | Frameshift | Ⅲ |
|  | *FKBP10* | INTRON5/  EXON 5 | c.918-3C>G/  c.879C>G | p.Tyr293* | Splicing/  Nonsense | Ⅳ |
|  | *FKBP10* | EXON 5 | c.831dupC | p.Gly278Argfs*95 | Frameshift | Ⅳ |
|  | *FKBP10* | EXON 5/  EXON 9 | c.764_772dupACGTCCTCC/c.1405G>T | p.255_257dupHisValLeu/p.Gly469X | Frameshift/  Nonsense | Ⅲ |
|  | *FKBP10* | INTRON 5/  EXON 6 | c.917+9G>A/c.955_975dupCAGGGTTACATCATCCCCGGG | p.Gly325_Met326insQGYIIPG | Splicing/  Frameshift | Ⅳ |
|  | *FKBP10* | INTRON 5/  EXON 5 | c.831_832insC/  c.918-6T>G | p.Gly278Argfs*95 | Frameshift/  Splicing | Ⅲ |
|  | *FKBP10* | EXON 7 | c.1160G>A | p.Arg387Gln | Missense | Ⅰ |
|  | *FKBP10* | EXON 8/  INTRON 5 | c.1270delG/  c.918-3C>G | p.Ala424Profs*12 | Frameshift/  Splicing | Ⅳ |
|  | *FKBP10* | EXON 8/  INTRON 5 | c.1270delG/  c.918-3C>G | p.Ala424Profs*12 | Frameshift/  Splicing | Ⅰ |
|  | *TMEM38B* | EXON 4 | c.507G>A | p.Trp169* | Nonsense | Ⅳ |
|  | *TMEM38B* | EXON 4 | c.507G>A | p.Trp169* | Nonsense | Ⅳ |
|  | *TMEM38B* | EXON 4 | c.507G>A | p.Trp169* | Nonsense | Ⅳ |
|  | *TMEM38B* | INTRON 3 | c.455-7T>G |  | Splicing | Ⅳ |
|  | *PLOD2* | EXON 18/  EXON 11 | c.2038C>T/  c.1138C>T | p.Arg659*/  p.Arg380Cys | Nonsense/  Missense | Ⅰ |
|  | *PLOD2* | INTRON 4/  EXON 11 | c.503-2A>G/  c.1138C>T | p.Arg380Cys | Splicing/  Missense | Ⅲ |
|  | *PLOD2* | EXON 14/  EXON 17 | c.1624_1624delT/  c.1880T>C | p.Tyr542Thrfs*18/  p.Val627Ala | Frameshift/  Missense | Ⅲ |
|  | *PLOD2* | EXON 18 | c.1856G>A | p.Arg619His | Missense | Ⅳ |
|  | *WNT1* | EXON 3 | c.506dupG | p.Cys170Leufs*6 | Frameshift | Ⅲ |
|  | *WNT1* | EXON 3/  EXON 3 | c.385G>A/c.506G>A | p.Ala129Thr/  p.Gly169Asp | Missense | Ⅳ |
|  | *WNT1* | EXON 3/  EXON 3 | c.506_507insG/  c.506G>A | p.Cys170Leufs*6/  p.Gly169Asp | Frameshift/  Missense | Ⅳ |
|  | *WNT1* | EXON 3/  EXON 3 | c.385G>A/  c.610G>T | p.Ala129Thr/  p.Glu204* | Missense/  Nonsense | Ⅲ |
|  | *WNT1* | EXON 3/  EXON 4 | c.502G>A/  c.677C>T | p.Gly168Arg/  p.Ser226Leu | Missense | Ⅲ |
|  | *WNT1* | EXON 3 | c.506_507insG | p.Cys170Leufs*6 | Frameshift | Ⅰ |
|  | *WNT1* | INTRON1/  EXON 3 | c.104+1G>A/  c.506G>A | p.Gly169Asp | Splicing/  Missense | Ⅰ |
|  | *WNT1* | EXON 4 | c.677C>T | p.Ser226Leu | Missense | Ⅰ |
|  | *WNT1* | EXON 4 | c.677C>T | p.Ser226Leu | Missense | Ⅰ |
|  | *WNT1* | EXON2/  EXON 3 | c.110T>C/  c.505G＞T | p.Ile37Thr/  p.Gly169Asp | Missense | Ⅳ |
|  | *BMP1* | INTRON 1/  EXON 11 | c.148+1G>A/  c.1324G>T | p.Asp442Tyr | Splicing/  Missense | Ⅳ |
|  | *P3H1* | EXON 14/  EXON 13 | c.2041C>T/  c.1873G>A | p.Arg681*/  p.Gly625Arg | Nonsense/  Missense | Ⅰ |
|  | *P3H1* | EXON 1 | c.81G>C | p.Glu27Asp | Missense | Ⅰ |
|  | *P3H1* | INTRON6/  EXON 9 | c.1170+5G>C/  c.1448G>A | p.Cys483Tyr | Splicing/  Missense | Ⅰ |
|  | *P3H1* | EXON 14/  EXON 13 | c.2041C>T/  c.1873G>A | p.Arg681*/  p.Gly625Arg | Nonsense/  Missense | Ⅰ |
|  | *P3H1* | EXON 14 | c.2387T>C | p.Leu796Pro | Missense | / |
|  | *CRTAP* | EXON 1 | c.451delC | p.Leu151Cysfs*23 | Frameshift | Ⅳ |
|  | *CRTAP* | INTRON 6/  INTRON 2 | c.1153-3C>G/  c.621+1G>A | / | Splicing | Ⅲ |
|  | *CRTAP* | EXON 7 | c.1153-3C＞G | / | Splicing | Ⅰ |
|  | *SEC24D* | INTRON 11 | c.1422-9_1422-8insT | / | Splicing | Ⅰ |
