# Supplementary material for: Genotype–phenotype relationship and comparison between eastern and western patients with osteogenesis imperfecta
Source: J Endocrinol Invest. 2023 Jun 4;47(1):67–77. doi: 10.1007/s40618-023-02123-2 (PMC10776744; doi:10.1007/s40618-023-02123-2)
Supplement: Supplementary file 3 — Supplementary file3 (DOCX 14 KB) [file 40618_2023_2123_MOESM3_ESM.docx]

Supplementary Table 2. Serum levels of 25OHD and PTH by quartiles of ages

|  | < 4.0 years  (n=79) | 4.0–10.0 years  (n=92) | 10.0–16.0 years  (n=88) | ≥ 16.0 years  (n=90) |
| --- | --- | --- | --- | --- |
| Serum 25(OH)D, ng/mL | 34.7 (24.4–47.5) | 23.0 (18.1–28.6) | 15.7 (12.6–22.5) | 15.7 (11.5–20.5) |
| Serum PTH, pg/mL | 18.1 (11.8–25.5) | 23.3 (15.9–31.0) | 24.8 (17.6–36.5) | 37.9 (27.9–49.7) |

Data were given as the median (range)
